# H3K18 lactylation marks tissue-specific active enhancers

**DOI:** 10.1186/s13059-022-02775-y

**Published:** 2022-10-03

**Authors:** Eva Galle, Chee-Wai Wong, Adhideb Ghosh, Thibaut Desgeorges, Kate Melrose, Laura C. Hinte, Daniel Castellano-Castillo, Magdalena Engl, Joao Agostinho de Sousa, Francisco Javier Ruiz-Ojeda, Katrien De Bock, Jonatan R. Ruiz, Ferdinand von Meyenn

**Affiliations:** 1 Laboratory of Nutrition and Metabolic Epigenetics, Institute for Food, Nutrition and Health, Department of Health Sciences and Technology, ETH Zurich, Zurich, Switzerland; 2 Functional Genomics Center Zurich, ETH Zurich and University Zurich, Zurich, Switzerland; 3 Laboratory of Exercise and Health, Institute of Human Movement Sciences and Sport, Department of Health Sciences and Technology, ETH Zurich, Zurich, Switzerland; 4 RG Adipocytes and Metabolism, Institute for Diabetes and Obesity, Helmholtz Diabetes Center at Helmholtz Center Munich, Neuherberg, 85764 Munich, Germany; 5 Department of Biochemistry and Molecular Biology II, School of Pharmacy, University of Granada, 18071 Granada, Spain; 6 PROFITH (PROmoting FITness and Health through Physical Activity) Research Group, Department of Physical Education and Sport, Faculty of Sport Sciences, University of Granada, Granada, Spain

**Keywords:** Lactylation, H3K18la, Lactate, Epigenetics, Embryonic stem cell, Muscle, Macrophage, Adipocyte, CUT&Tag, ChromHMM, Histone post-translational modification, Promoter, Enhancer

## Abstract

**Background:**

Histone lactylation has been recently described as a novel histone post-translational modification linking cellular metabolism to epigenetic regulation.

**Results:**

Given the expected relevance of this modification and current limited knowledge of its function, we generate genome-wide datasets of H3K18la distribution in various in vitro and in vivo samples, including mouse embryonic stem cells, macrophages, adipocytes, and mouse and human skeletal muscle. We compare them to profiles of well-established histone modifications and gene expression patterns. Supervised and unsupervised bioinformatics analysis shows that global H3K18la distribution resembles H3K27ac, although we also find notable differences. H3K18la marks active CpG island-containing promoters of highly expressed genes across most tissues assessed, including many housekeeping genes, and positively correlates with H3K27ac and H3K4me3 as well as with gene expression. In addition, H3K18la is enriched at active enhancers that lie in proximity to genes that are functionally important for the respective tissue.

**Conclusions:**

Overall, our data suggests that H3K18la is not only a marker for active promoters, but also a mark of tissue specific active enhancers.

**Supplementary Information:**

The online version contains supplementary material available at 10.1186/s13059-022-02775-y.

## Background

Histone modifications regulate DNA accessibility, chromatin structure and dynamics, and gene expression [1]. They can promote chromatin relaxation and gene transcription, or chromatin condensation and gene repression, respectively [2]. Since the initial discovery of acetylation and methylation of histones in 1964 [3], the number of described histone post-translational modifications (hPTMs) has significantly increased [4]. In 2019, lactylation of lysine residues of histones (Kla) was described for the first time [5]. Similar to histone acetylation and other histone acylation moieties [6], histone lactylation links (cellular) metabolism to epigenetic gene regulation. Indeed, both extracellular and endogenous lactate increase global histone lactylation levels while inhibition of glycolysis (and thus lactate production) reduces histone lactylation levels [5]. Glycolysis is a central energy producing process, and consequently, lactate is produced (and consumed) in almost all cellular systems and mammalian tissues [7]. Besides representing the end-product of glycolysis, lactate is also the main circulating metabolite that feeds into the tricarboxylic acid (TCA) cycle [8], an important signaling molecule, and a major substrate for gluconeogenesis [9]. Because of lactate’s omnipresence, histone lactylation may be present in all mammalian systems, but this remains to be verified. More than 30 histone lactylation sites have been reported in human, mouse, plant, fungal, and parasitic (protozoic) samples [5, 10–20]. Lactylation of histone 3 on lysine residue 18 (H3K18la) has been studied in more detail and shown to be highly enriched on gene promoters and to correlate with active gene expression of the associated genes in cancer cells and in macrophages [5, 17]. Hitherto, most reports studying Kla focused on changes in total Kla levels, but the genome-wide H3K18la distribution and its relation to other histone modifications and gene expression are poorly described.

Here, we show that H3K18la is present in a broad range of human and mouse cell types and tissues. Most importantly, we report that H3K18la is not only enriched at promoters, but also at active enhancers in a tissue-specific manner, and resembles, although does not copy, H3K27ac genomic localization.

## Results

### Histone lactylation is present in tissues representing a broad range of metabolic states

To explore the role of H3K18la, we investigated its genome-wide localization in a broad panel of in vitro and in vivo samples. We selected samples that differ in developmental stage and mitotic activity, since histone lactylation has been correlated to glycolytic activity and lactate levels [5], and that span a broad metabolic range with differing intracellular lactate levels. We included mouse embryonic stem cells, cultured in conditions that recapitulate embryonic naïve (mESC-2i; grown in 2i LIF) or primed pluripotency states (mESC-ser; grown in serum LIF) [21] and also differ in their metabolic status [22]. Indeed, highly glycolytic mESC-ser have higher lactate levels compared to mESC-2i (Supplementary Figure 1A). We also included primary muscle stem cells (myoblasts, MB) and in vitro differentiated multinucleated post-mitotic end-state myotubes (MT), as well as in vivo mouse muscle samples (gastrocnemius, GAS). MT display higher OXPHOS and similar glycolytic rates compared to MB [23]. Nevertheless, MT have higher lactate levels compared to MB (Supplementary Figure 1A and [24]). Similarly, muscles are known to be metabolically highly active and producing high amounts of lactate [25]. In contrast to muscle tissue, we included adipocytes from white epididymal adipose tissue (ADIPO), which is known for its particularly low metabolic rate [26]. MB, MT, GAS, and ADIPO are all cell types/tissues originating from the mesenchymal cell lineage. Lastly, we included bone marrow-derived macrophages (BMDM), in which histone lactylation was originally described [5], as well as macrophages that are recruited to the muscle after induction of tissue ischemia (see the “Materials and methods” section, “post-ischemia macrophages,” PIM). BMDMs and PIMs were shown to respond to exogeneous lactate by upregulating anti-inflammatory gene signatures [5, 27], which was shown to be partly due to hyperlactylation of the affected genes’ promoters in BMDMs. Noteworthy, during tissue repair and associated macrophage polarization, macrophages undergo a dramatic metabolic shift which is required for their phenotypic shift [28].

**Fig. 1:**
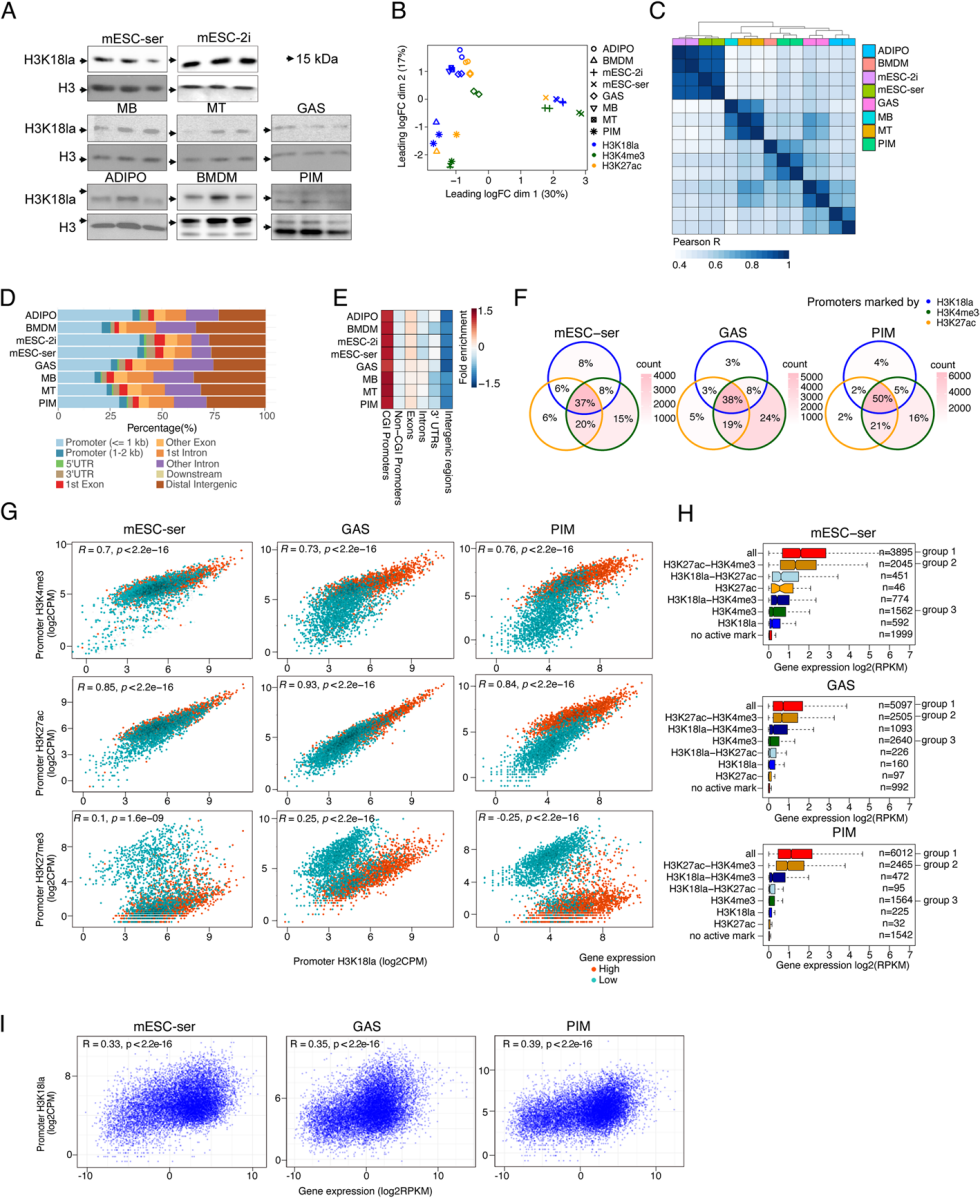
H3K18la marks active CGI promoters. **A** Western blots showing H3K18la and H3 protein expression in all included samples (*n* = 3). The arrows indicate 15 kDa. **B** MDS of active hPTMs profiled from various mouse samples, quantified over 3000 bp genome-wide tiles. **C** Correlation heatmap of genome-wide, quantified H3K18la peak levels (biological replicates (*n* = 2) for mESC-ser, mESC-2i, ADIPO, GAS, PIM, and MT. For MB and BMDM; *n* = 1. Pearson’s correlation coefficient *R* is displayed as color gradient. **D** Distribution of H3K18la peaks of all mouse samples across genomic features. **E** H3K18la peak fold enrichment adjusted for genome-wide feature size for all mouse samples. **F** Venn diagrams depicting the promoter overlaps marked by the active hPTMs in mESC-ser, GAS, and PIM samples. Overlaps are colored according to the absolute number of promoters marked by various combinations of active hPTMs. Percentages indicate the fraction of actively marked promoters belonging to each group. **G** Scatterplots showing pairwise correlation of promoter H3K18la levels with other hPTM levels (log_2_CPM) highlighting the promoters of genes with highest (red, *n* = 2000) or lowest (cyan, *n* = 2000) normalized gene expression (RPKM) for mESC-ser, GAS, and PIM. Pearson’s correlation coefficient *R* and *p*-values are indicated. **H** Normalized gene expression (log_2_RPKM) per gene category is shown as boxplots. Gene categories are defined by the combination of active hPTMs as **F**. **I** Scatter plots showing the correlation between promoter H3K18la levels (log_2_CPM, *y*-axis) and expression of the corresponding gene (log_2_RPKM, *x*-axis) for mESC-ser, GAS, and PIM. Spearman’s correlation coefficient *R* and *p*-values are indicated

Western blotting showed that H3K18 lactylation is present in all cells and tissues included in this study (Fig. 1A, Additional file 2). To investigate whether changes in cellular metabolism, and thus intracellular lactate levels, affect global H3K18la, we compared H3K18la levels in related cell pairs: MB versus MT and mESC-ser versus mESC-2i. We found no correlation between intracellular lactate levels and H3K18la or panKla levels, except for panKla in mESC (Additional file 1: Fig. S1B, Additional file 2). However, when we added 10mM sodium-L-lactate to the cell medium, H3K18la as well as panKla levels did increase in all cases (Additional file 1: Fig. S1B, Additional file 2), as has been shown previously for other cell types [5]. This suggests that global H3K18la levels are not directly linked to (small) metabolic differences between cell types. However, when stimulated with a large lactate surplus, H3K18la levels do increase. This is in line with data presented by Zhang et al. [5], where such large lactate changes were studied.

### H3K18la marks active promoters

To study the functional role of H3K18la, we generated CUT&Tag sequencing libraries [29] for H3K18la and additional active (H3K4me3 and/or H3K27ac) and repressive (H3K27me3) hPTMs allowing us to profile their genomic localization. All datasets were processed, quality checked, and mapped using standardized pipelines (Additional file 1: Fig. S1C), and the quality metrics have been summarized in Additional file 3: Table S1.

We first explored how the datasets compare to each other globally. To this end, we quantified all samples over genome-wide tiles spanning 3000 bp each and performed multidimensional scaling (MDS) analysis. The samples clustered based on the type of mark (active versus repressive; Additional file 1: Fig. S1D) and according to the origin of the sample and specific hPTM profiled (Fig. 1B). mESC displayed the most distinct profiles from differentiated tissues, especially for active marks (Additional file 1: Fig. S1D). MB, MT, and GAS active marks clustered together as well as BMDM and PIM datasets (Fig. 1B). For each cell type, H3K18la samples clustered closer to H3K27ac than to H3K4me3 samples (Fig. 1B). This highlights that the genomic distribution of H3K18la is tissue-specific, resembles H3K27ac from the same tissue-type, and is retained in vitro. Of note, H3K18la ADIPO samples clustered closest to muscle sample despite being characterized by significantly differing metabolic rates. This might reflect their similar developmental programs and mesenchymal lineage origins. Despite differences in metabolic status between mESC-2i and mESC-ser, or MB and MT, their H3K18la profiles also clustered based on their origin. These results indicate that developmental identity is important for H3K18la genomic distribution.

Next, we used the SEACR peak caller [30] to define hPTM enrichment. We observed that SEACR often called multiple nearby smaller peaks together (Additional file 1: Fig. S1E). A correlation analysis of the quantified H3K18la peaks confirmed that the mESC H3K18la profiles were most distinct from the differentiated cell types (Fig. 1C). Macrophages (BMDM and PIM) were closely correlated as well as in vitro (MB and MT) to in vivo muscle samples (GAS), and tissues from the mesenchymal lineage (ADIPO and GAS), corroborating H3K18la profiles’ cell type-specificity (Fig. 1B). In accordance with data published by Zhang et al*.* [5], we found many H3K18la peaks localized near transcription start sites (TSS) and overlapping with gene promoters or introns (Fig. 1D and Additional file 1: Fig. S1F-G). The fraction of H3K18la peaks within promoter regions was highest in mESC and ADIPO (~40%) (Fig. 1D). H3K18la peak distribution of ADIPO, GAS, PIM, MB, and MT were slightly shifted downstream of the TSS, which was also true for the corresponding H3K4me3/H3K27ac active marks, but not for (repressive) H3K27me3 peaks (Additional file 1: Fig. S1G). Size-corrected enrichment analysis for genomic features showed distinct enrichment of H3K18la signal in CpG island (CGI) promoters but not in non-CGI promoters (Fig. 1E).

To investigate if promoters can be marked by different combinations of active hPTMs, we overlapped the promoters marked by H3K4me3, H3K27ac, and/or H3K18la peaks (Fig. 1F). We found that the vast majority of these “active” promoters are either marked by H3K4me3+H3K27ac+H3K18la (37–50%), by H3K4me3+H3K27ac (19–21%), or by H3K4me3 alone (15–24%). Therefore, only about half of all H3K4me3-marked promoters are also marked by H3K18la. The correlation of promoter H3K4me3 to H3K18la levels confirmed a subset of gene promoters with high H3K4me3 levels, but not high H3K18la levels (Fig. 1G, Additional file 1: Fig. S2A). This subset was not prominent for CGI promoters (Additional file 1: Fig. S2B). To investigate if there is a functional difference between H3K18la-marked active promoters and non-H3K18la-marked active promoters, we looked at gene expression, Gene Ontology (GO) enrichment, and TF-binding site enrichment of their associated genes. Firstly, genes with H3K18la+H3K4me3+H3K27ac-marked promoters (group 1) were significantly higher expressed than genes with H3K4me3+H3K27ac-marked promoters (group1 versus group 2, *p* < 3.3×10e−7) or H3K4me3-only-marked promoters (group1 versus group 3, *p* < 2.2×10e−16) (Fig. 1H). In fact, there is an additive effect of promoter H3K18la- and/or H3K27ac-marking on gene expression of genes (or vice versa), with that of H3K27ac being considerably bigger than that of H3K18la. This indicates that H3K18la primarily marks the promoters of the highest expressed genes. Also at a quantitative level, H3K18la promoter levels did correlate positively with gene expression in all samples (Fig. 1I). H3K18la promoter levels at CGI promoters correlated even better with the expression of their associated genes (Additional file 1: Fig. S2C). Further, group 1 (promoters marked by H3K18la+H3K4me3+H3K27ac) genes are most strongly enriched in tissue-specific gene ontology (GO) terms, especially for PIM and GAS. Group 2 or group 3 genes (no promoter H3K18la) are more distinctive for RNA- and ribosome-related terms (Additional file 1: Fig. S3A). Lastly, differentially marked promoters were analyzed in Cistrome [31] to discover whether they were enriched for different TF-binding sites. The biggest difference was observed between group 3 promoters versus group 1/2 promoters. The group 3 promoter coordinates were generally most similar to binding patterns of repressive TFs related to PRC2, such as JARID2, MTF2 SUZ12, RNF2, and EZH2, while group 1/2 promoter sets were most similar to H2AZ positioning, POLR2A and KMT2C binding (Additional file 1: Fig. S3B). Confirming their poised state, 45% or more of group 3 promoters were also marked by H3K27me3 peaks and this was not the case for group 1/2 promoter sets (Additional file 1: Fig. S3C).

In summary, we found that H3K18la is enriched in a subset of (primarily CGI) active gene promoters, and H3K18la promoter levels correlate positively with gene expression and the well-established active marks H3K4me3 and H3K27ac.

### H3K18la marks active enhancers

**Fig. 2:**
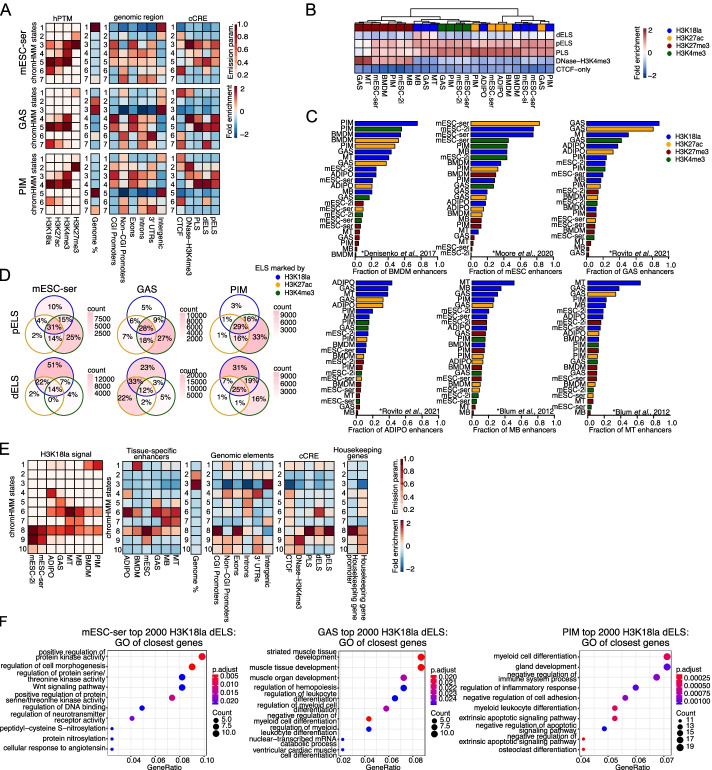
H3K18la marks active, tissue-specific enhancers. **A** Tissue- and cell-type-specific ChromHMM analysis of mESC-ser, GAS, and PIM based on their hPTM profiles. The color scale corresponds to the emission parameter of each hPTM for each state. Fold enrichment of ChromHMM states for total genomic fraction coverage, genomic features, and ENCODE cCREs, scaled from −2 to 2 (see the “Materials and methods” section for details). **B** Heatmap showing the fold enrichment of hPTM peaks in ENCODE cCREs ; Σ (bp overlap)/[Σ (bp PTM peak)*Σ (bp cCRE)], scaled from −2 to 2. **C** Bar plots depicting the fraction of published tissue-specific enhancers [34–37] that overlap with hPTM peaks. **D** Venn diagrams depicting the overlap of pELS/dELS marked by the active hPTMs in mESC-ser, GAS, and PIM samples. Overlaps are colored according to the absolute number of ELS marked by various combinations of active hPTMs. Percentages indicate the fraction of actively marked ELS belonging to each group. **E** ChromHMM analysis of all tissues/cell types based on their H3K18la profiles. The color scale shows the emission parameter of each tissue/cell type for each state. Fold enrichment of ChromHMM states over published tissue-specific enhancer sets [34–37], total genomic fraction coverage, genomic features, ENCODE cCREs, house-keeping gene promoters, and house-keeping genes [38], scaled from −2 to 2 (see the “Materials and methods” section for details). **F** Top 10 GO (category “Biological Process”) terms resulting from the GO enrichment analysis of the genes closest to the top 2000 dELS from ENCODE cCRE with highest H3K18la levels (see the “Materials and methods” section for how dELS were linked to genes)

While H3K18la peaks were strongly enriched around TSS, we also observed a substantial fraction of H3K18la peaks distal from TSS (>2 kb) (Additional file 1: Fig. S1G), and/or not overlapping with promoter regions, but instead localized at intronic or intergenic regions (Additional file 1: Fig. S1F). The fraction of H3K18la peaks in intronic regions was highest in the differentiated cell types and lowest in mESC. Interestingly, enhancers with tissue-specific activity were recently reported to be enriched in intronic regions [32]. Moreover, our genome-wide correlation analyses uncovered that H3K18la resembles H3K27ac (typical marker for active promoters and active enhancers) more than H3K4me3 (typical marker for active promoters but not enhancers). To confirm that H3K18la marks active enhancers, we performed an unsupervised ChromHMM [30] analysis which allowed us to estimate genome-wide co-occurrence of H3K18la with H3K27ac with or without H3K4me3. ChromHMM [33] is based on a multivariate hidden Markov model and integrates multiple datasets to discover the major re-occurring combinatorial and spatial patterns in the genome. For all three investigated samples (mESC-ser, GAS, and PIM), we defined 7 ChromHMM states (see Materials and methods), 3 of which were marked by different combinations of active marks: H3K4me3+H3K27ac+H3K18la, H3K4me3+H3K27ac, and H3K27ac+H3K18la (Fig. 2A). H3K18la did not co-occur with H3K4me3 without H3K27ac and neither H3K18la nor H3K4me3 occurred without H3K27ac. The H3K4me3+H3K27ac+H3K18la and H3K4me3+H3K27ac states displayed similar enrichment over genomic elements. The H3K27ac+H3K18la state was enriched in introns and non-CGI-promoters and particularly depleted in CGI promoters. To investigate whether this state potentially represents enhancer regions, we calculated ChromHMM state enrichment over ENCODE’s database of cell type agnostic candidate cis-regulatory elements (cCRE) [34]. This database contains genomic coordinates of promoter-like sequences (PLS, defined as: <200 bp from TSS and marked by H3K4me3) and enhancer-like sequences (ELS), subdivided into proximal enhancer-like sequences (pELS, defined as: between 200 and 2000 bp from TSS and marked by H3K27ac; of note this definition can overlap with promoters) and distal enhancer-like sequences (dELS, defined as: >2000 bp from TSS and marked by H3K27ac). Confirming our hypothesis, the H3K27ac+H3K18la state was enriched in dELS. In fact, every dELS enriched ChromHMM state was always marked by H3K18la. Besides dELS, the H3K27ac+H3K18la state was also strongly enriched in CTCF-binding sites. When overlapping peaks with cCRE (see the “Materials and methods” section), we observed that both H3K18la and H3K27ac peaks were enriched at dELS (Fig. 2B), which was not true for H3K4me3, which was primarily enriched at PLS and pELS. Of note, the ENCODE cCRE database does not include repressed regions, and as such, the absolute enrichment of H3K27me3 peaks across cCRE was low. For the few H3K27me3 peaks that did overlap with cCRE, we found them at PLS, pELS, or non-promoter regions marked by Dnase/H3K4me3.

Encouraged by these findings, we next compared our hPTM profiles with public ChIP-seq datasets [5, 34, 35, 39–41]. We included public datasets (matching our tissues) from hPTMs commonly used to identify enhancers, i.e., H3K4me1 and H3K27ac (active enhancers only [42]). We also included H3K4me3, H3K27me3, and H3K18ac for their potential similarity to H3K18la and association with enhancers [43]. Lastly, we included the only other available genome-wide H3K18la profiles from BMDM [5]. Reassuringly, our CUT&Tag datasets overlapped well with the public ChIP-seq datasets (Additional file 1: Fig. S4A). Public BMDM H3K18la and H3K18ac datasets showed high correlation with our H3K18la BMDM profiles (H3K18ac and H3K18la have also been shown to correlate globally by Zhang et al. [5]). Further, our mESC H3K18la peaks overlapped well with public H3K27ac, H3K4me1, and H3K4me3 peaks from mESC, and we made similar observations for the other tissues. This is consistent with our observation that H3K18la marks active promoters as well as active enhancers, which are both typically marked by H3K27ac. The stronger overlap with H3K27ac as compared to H3K4me1 suggests that H3K18la is marking active enhancers and not poised/inactive enhancers [42]. Remarkably, our MT H3K18la profiles overlapped best with public GAS H3K27ac profiles, followed by public MT and MB H3K18ac profiles, indicating a good overlap between the epigenomes of our primary in vitro differentiated MTs and those of mouse muscle. Together, the overlap between our H3K18la profiles and public tissue-specific ChIP-seq datasets supports the notion that H3K18la marks active (and not poised/inactive), tissue-specific enhancers.

To further validate these results, we obtained tissue-specific enhancer tracks from literature [34–37, 44] and calculated which fraction of these enhancers overlap with H3K18la peaks. Notably, for 5 out of the 7 investigated tissues (not for published MB and ADIPO enhancers), more than 60% of published tissue-specific enhancers were covered by our tissue-corresponding H3K18la peaks (Fig. 2C). Since the ADIPO enhancers were defined based on results from whole adipose tissue and not sorted adipocytes as were used in this study, there may be many enhancers that are not specific to adipocytes but rather to other adipose-tissue-resident cells. Moreover, for most published tissue-specific enhancers, none of our other peak sets outcompetes the matching tissue-specific H3K18la peaks. One of the two exceptions is our mESC-ser H3K27ac peak set, which covers slightly more E14 enhancers than our mESC-ser and mESC-2i H3K18la peak sets. The other is our MT’s H3K18la peak set that covers a larger fraction (~55%) of published MB-specific enhancers than our MB’s H3K18la peak set. The published dataset is based on the C2C12-cell line, while our data originates from primary myoblasts, which may explain the discrepancy. Notably, our PIM’s H3K18la peaks cover public BMDM enhancers better than our BMDM H3K18la/H3K27ac or PIM H3K27ac peaks. Since most studies use H3K27ac occupancy as a defining criterium for enhancer identification, we investigated which fraction of cell-type agnostic ELS was covered by a combination of H3K18la, H3K27ac, and/or H3K4me3 peaks (Fig. 2D). To our surprise, we found that a substantial fraction of putative dELS was marked only by H3K18la peaks but not by H3K27ac peaks (or H3K4me3), suggesting additional H3K18la-specific roles in dELS.

Next, we used all our H3K18la datasets to generate a ChromHMM model based on 10 chromatin states (Fig. 2E). As opposed to its classical use (multiple different hPTMs in 1 sample, as used above), we here employ the method in an alternative way (1 hPTM in multiple different samples) to discover H3K18la-marked genomic regions in a more tissue/cell type-specific and agnostic manner. Five out of these 10 chromatin states were tissue-type specific, indicating that the annotated genomic regions are defined by the H3K18la levels of the respective tissue, i.e., state 1: macrophage (BMDM+PIM); state 4: ADIPO; state 5: GAS; state 7: MB+MT; and state 9: mESC. Strikingly, the tissue-specific states were without exception found to be enriched for matching published tissue-specific enhancers (Fig. 2E) as well as for matching tissue-specific marks for active enhancers (Additional file 1: Fig. S4B). State 6 and state 8 annotate genomic regions defined by H3K18la levels across all differentiated sample types and all samples, respectively. State 6 (shared across all differentiated cell types) was strongly enriched in dELS, while state 8 (shared across all cell types) was strongly enriched in PLS, pELS, exons, and CGI promoters. CGIs are known to be enriched in promoters of house-keeping genes, and less in promoters of tissue-specific genes [45–47]. Indeed, state 8 was strongly enriched in housekeeping gene promoter regions (housekeeping genes as defined in [38]) (Fig. 2D), including those of *Gapdh*, *Actb*, *B2m*, *Ubc*, *Pgk1*, *Ppia*, *Ywhaz*, *Rpl13a*, and *Tfrc* (Additional files 4 and 5: Tables S2-3)*.* This ChromHMM analysis confirmed that H3K18la localization is highly tissue-specific, marking enhancers that are active in the investigated tissue. H3K18la also marks active CGI promoters that are broadly shared between different tissues and marked by active hPTMs in various tissue types. Concordant with these findings, many of these CGI promoters are associated to housekeeping/constitutively expressed genes.

We then set out to investigate whether enhancers marked by H3K18la peaks are related to higher expression of target genes. We linked each dELS to its nearest but not overlapping promoter of a protein-coding-gene and calculated the correlation between H3K18la dELS levels and the expression of its putative linked gene. Although weak, the correlation between dELS H3K18la peak levels and expression of their nearest gene was positive and significant for all samples (Additional file 1: Fig. S3C). The genes closest to the 2000 dELS with the highest H3K18la peaks were strongly enriched in several tissue-specific GO-categories (Fig. 2F, top 10 GO terms).

In conclusion, besides CGI promoters of highly expressed genes, including both constitutively expressed housekeeping genes and tissue-specific genes, H3K18la marks active enhancers in a tissue-specific manner. Moreover, genes that lie closest to enhancers marked by high levels of H3K18la are important for tissue-specific gene expression and enhancer H3K18la levels correlate weakly, though significantly, with the expression of their nearest genes.

### Dynamic changes of H3K18la reflect transcriptional adaptations

**Fig. 3:**
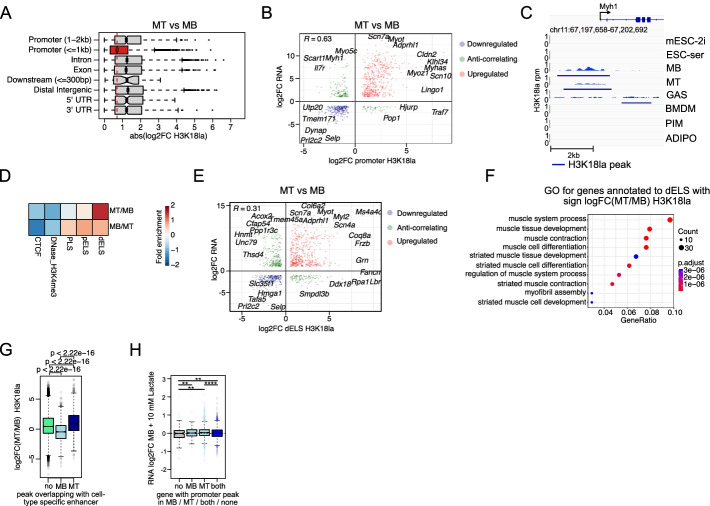
Dynamic changes of H3K18la reflect transcriptional adaptations. **A** Box plots showing H3K18la log_2_FC changes from MT versus MB over different genomic features. **B** Scatterplot showing the correlation between significant (FDR < 0.05) H3K18la log_2_FC (>0.5) in promoters and their corresponding gene expression log_2_FC (>0.5) based on the overlapping genes from MT versus MB differential analysis. Pearson correlation coefficient *R* is indicated. **C** IGV genome browser [50] snapshot of H3K18la profile at the *Myh1* promoter region from various mouse samples and the corresponding SEACR-called peak regions. H3K18la level is depicted (rpm). Genomic regions are indicated on the top, as well as RefSeq gene names. **D** Fold enrichment of significant differentially H3K18la-marked peaks (FDR < 0.05, |log_2_FC| > 1.5) in ENCODE cCREs. **E** Scatterplot showing the correlation between significant (FDR < 0.05) H3K18la log_2_FC (>0.5) in dELS and their closest gene expression log_2_FC (>0.5) based on the overlapping genes from MT versus MB differential analysis. Pearson correlation coefficient *R* is indicated. **F** Top 10 GO terms (category “Biological Process”) based on the GO analysis of the overlapping upregulated genes in MT from **E** (first quadrant red dots). **G** Box plots showing H3K18la log_2_FC of peaks overlapping with MB- or MT-specific enhancers and of peaks not overlapping with these enhancers. Wilcoxon test *p*-values are indicated for each pair of groups. **H** Gene expression changes (log_2_FC) after treatment of MB with 10 mM sodium-L-lactate of genes containing a H3K18la peak in MB, in MT, in both or in none of both. Wilcoxon test *p*-values are indicated for each pair of groups. *p value < 0.05, **p value < 0.01, ***p value < 0.001, ****p value < 0.0001

We next focused on putative functionally relevant changes in H3K18la between closely related cell types and compared MT versus MB and mESC-ser versus mESC-2i. For each pair, we computed a union peak set (see the “Materials and methods” section) and quantified the regions. Peaks overlapping with (core) promoters were more stable than peaks in other genomic regions (Fig. 3A, Additional file 1: Fig. S5A), confirming our prior results (ChromHMM state 8 enriched in promoters; Fig. 2E). Nonetheless, we also observed many promoters gaining or loosing H3K18la in both cell state transitions. Overall, these changes correlated positively with up- or downregulation, respectively, of their associated genes (*R* = 0.63 for MT/MB and *R* = 0.51 ser/2i) (Fig. 3B, Additional file 1: Fig. S5B), e.g., *Neurog3* in mESCs or *Myhas* in MT/MB (Additional file 1: Fig. S5C, Additional file 1: Fig. S1E). In addition, we observed that for a minority of genes, promoter lactylation changes, and gene expression changes did not positively correlate. One notable example is *Myh1* (Fig. 3B, C). Interestingly, *Myh1* expression is regulated by the fast myosin heavy chain super-enhancer (fMyh SE; Additional file 1: Fig. S1D), which is also strongly H3K18la marked. *Myhas*, also known as *Linc-Myh*, is adjacent to the fMyh SE and suppresses slow-type Myh gene expression while stimulating fast-type Myh gene expression (*Myh1*, *Myh2*, *Myh4*) through enhancer-promoter looping [48, 49]. It is therefore plausible that *Myh1* gene expression is regulated in MT through *Myhas* and activation and hyperlactylation of its enhancer.

In line with our prior observations, enhancer (dELS) lactylation is much more dynamic than H3K18la changes in any other genomic region (Fig. 3D, Additional file 1: Fig. S5D), and H3K18la changes in dELS do also positively correlate with changes in gene expression of the closest genes (Fig. 3E, Additional file 1: Fig. S5E). GO analyses of the genes in closest proximity to dELS with significant H3K18la changes in MT versus MB (Fig. 3F) or mESC-ser versus mESC-2i (Additional file 1: Fig. S5F) identified GO terms related to the respective cell types. A further detailed analysis of MB- and MT-specific enhancers [36] revealed that H3K18la occupancy changed accordingly in each dataset (Fig. 3G) and supports our hypothesis that quantitative lactylation changes at promoters and enhancers recapitulate and possibly even promote cell state transitions.

Lactate has been suggested to stimulate myogenic differentiation, including the transition from MB to MT [51–53], and indeed, many promoters and enhancers gain H3K18la during the MB to MT transition (Fig. 3B, E). We asked if lactate treatment of MB would be sufficient to upregulate the subset of genes that show high promoter lactylation in MT. Globally, the effect of lactate treatment on gene expression was minimal (Additional file 6: Table S4). Using less stringent criteria, we found a small group of differentially expressed genes (nom *P* < 0.01 abs(log2FC) > 0.5; upregulated *n* = 40; downregulated *n* = 19). The upregulated genes were related to “lactate metabolic process” and “positive regulation of striated muscle contraction” (GO enrichment analysis; FDR = 0.04 for both) and almost half (*n* = 18) of these genes had a MT-specific H3K18la promoter peak. Notably, the latter were enriched in several myogenesis-related GO terms (e.g., “skeletal muscle tissue development,” FDR = 0.014; “striated muscle cell differentiation,” FDR = 0.022). A parallel analysis showed that genes with an H3K18la promoter peak in MT were on average slightly upregulated in MB treated with 10 mM lactate (Fig. 3H), agreeing with the hypothesis that lactate could stimulate myogenic differentiation through increased promoter histone lactylation. Together, our data suggests that lactate may be a cell-state-transition stimulating metabolite through hyperlactylation of gene promoters and enhancers that are specific to the end-state. This hypothesis and data are in line with the results presented for promoter hyperlactylation in macrophage polarization by Zhang et al. and with the results by Yu et al. showing promoter lactylation stimulates oncogenesis in ocular melanoma [5, 17].

### H3K18la is conserved and marks active promoters and enhancers in human muscle

**Fig. 4:**
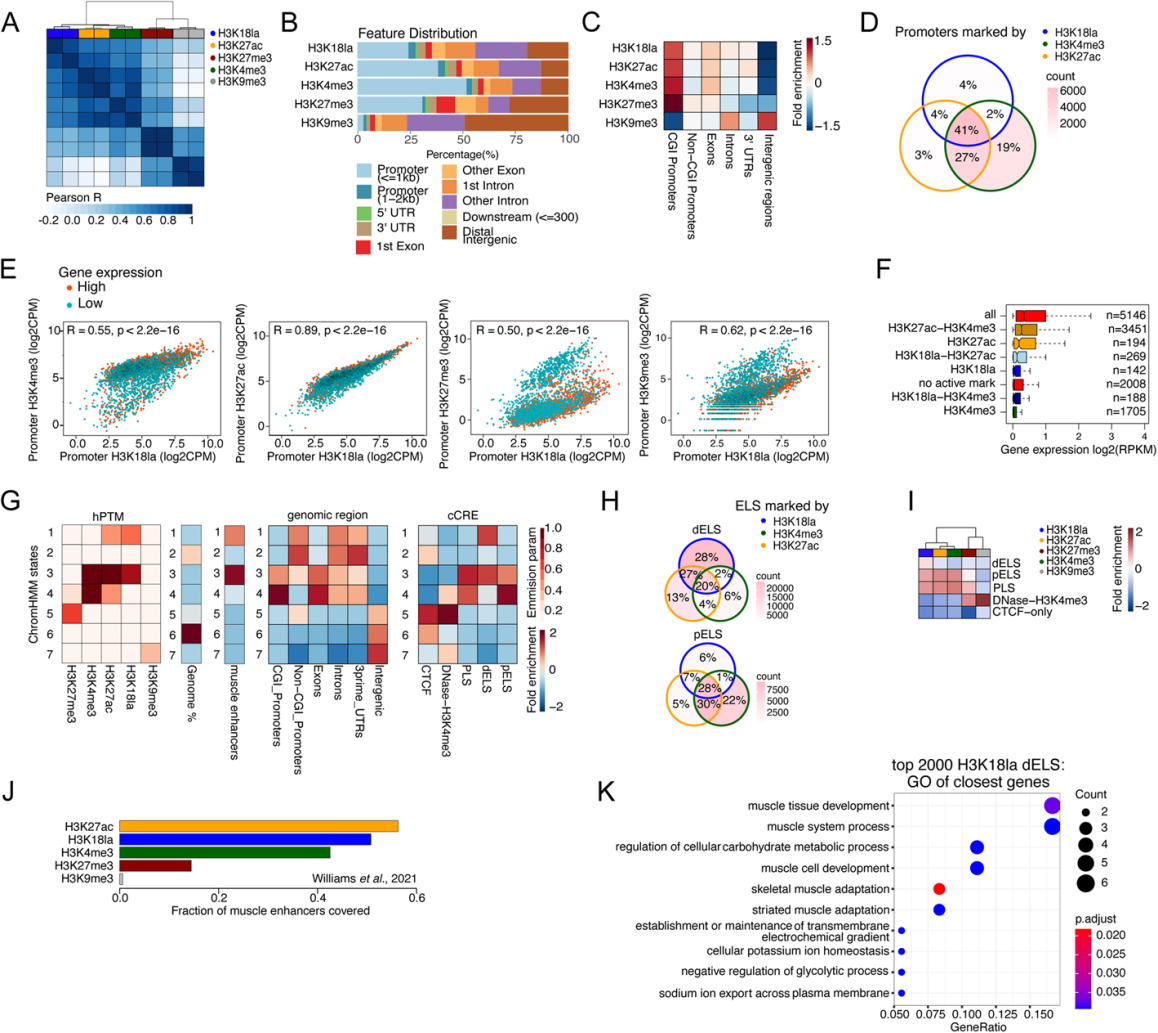
H3K18la in human muscle marks active promoters and enhancers. **A** Correlation heatmap of hPTM levels in genome-wide quantified peaks from human muscle. Pearson’s correlation coefficient *R* is displayed as color gradient. **B** Distribution of all hPTMs across genomic features. **C** Peak fold enrichment adjusted for genome-wide feature size. **D** Venn diagram depicting the promoter overlaps marked by the active hPTMs. Overlaps are colored according to the absolute number of promoters marked by various combinations of active hPTMs. Percentages indicate the fraction of actively marked promoters belonging to each group. **E** Scatterplots showing pairwise correlation of promoter H3K18la levels with other hPTM levels (log_2_CPM) highlighting the promoters of genes with highest (red, *n* = 2000) or lowest (cyan, *n* = 2000) normalized gene expression (RPKM). Pearson’s correlation coefficient *R* and *p*-values are indicated. **F** Normalized gene expression (log_2_RPKM) per gene category is depicted as boxplots. Gene categories are defined by the combination of active hPTMs as in **D**. **G** ChromHMM analysis based on 5 hPTMs profiled in human muscle. The color scale shows the emission parameter of each mark for each state. Fold enrichment of ChromHMM states for total genomic fraction coverage, published skeletal muscle enhancers [57], different genomic features, and ENCODE cCREs, scaled from −2 to 2. **H** Venn diagrams depicting the overlap of dELS and pELS as marked by the different combination of the active hPTMs. Overlaps are colored according to the absolute number of ELS marked by various combinations of active hPTMs. Percentages indicate the fraction of actively marked ELS belonging to each group. **I** Heatmap showing the fold enrichment of hPTM peaks in cCREs (ENCODE); Σ (bp overlap)/[Σ (bp PTM peak)*Σ (bp cCRE)], scaled from −2 to 2. **J** Bar plots depicting the fraction of published human muscle enhancers [57] overlapping with the human hPTM peaks. **K** Top 10 GO terms (category “Biological Process”) resulting from a GO analysis of the corresponding closest genes to the 2000 dELS with highest H3K18la peaks (see the “Materials and methods” section for details on how enhancer and gene were linked)

To confirm whether our findings are also conserved in human, we profiled the epigenome of the vastus lateralis muscle from two human subjects, assessing H3K18la, H3K27ac, H3K4me3, H3K27me3, and H3K9me3 (well-established mark for repressed, heterochromatic regions [54–56]). Multi-dimensional scaling analysis of quantified genome-wide 3000 bp tiles from all human muscle samples separated active from repressive marks, clustering specific types of marks closer together (Additional file 1: Fig. S6A). Compared to the mouse data, H3K27ac clustered between H3K18la and H3K4me3 on the first dimension (Additional file 1: Fig. S6A). We next called hPTM peaks as described above. In accordance with the MDS analysis, correlating all quantified peaks with each other showed that the H3K18la profiles correlated best with H3K27ac and H3K4me3 (Fig. 4A). The distribution of H3K18la peaks over different genomic features closely resembled the results from the mouse muscle samples (Figs. 1D and 4B), with H3K18la enriched at promoter regions, intronic regions, and intergenic regions (Fig. 4B and Additional file 1: Fig. S6B) and a substantial fraction of these peaks localized > 10 kb from the TSS (Additional file 1: Fig. S6C). H3K18la peaks were strongly enriched at CGI promoters and less in non-CGI-promoters (Fig. 4C). Most promoters marked by H3K18la were also marked by H3K27ac and H3K4me3 (Fig. 4D). H3K18la peak levels at gene promoter regions did correlate strongly to H3K27ac as well as to H3K4me3 peak levels (Fig. 4E). Genes with H3K4me3 marked promoters were higher expressed if they were also marked by H3K18la and/or H3K27ac (Fig. 4F). Overall, we found that the levels of H3K18la at promoters correlated rather weakly (though positively) to a public gene expression dataset from the same muscle [57], especially when compared to H3K4me3 or H3K27ac (Additional file 1: Fig. S6D). Nevertheless, GO analysis of the top 2000 genes with the highest promoter H3K18la levels (Additional file 1: Fig. S6E) showed that H3K18la marks promoters of genes important to muscle biology, whereas genes with active histone marks in their promoters (H3K27ac and/or H3K4me3) without H3K18la were not enriched for muscle biology terms (Additional file 1: Fig. S6F). H3K18la promoter levels of CGI promoters correlated stronger to the expression of their associated genes than when considering all promoters (Additional file 1: Fig. S6G). Likewise, the correlation between H3K18la levels and H3K27ac and H3K4me3 levels was higher for CGI promoters than for all promoters (Additional file 1: Fig. S6H).

We next used our set of human muscle hPTM profiles to perform an unbiased ChromHMM analysis of human muscle chromatin patterns. The results recapitulated the mouse ChromHMM analyses. We found that a model with 7 distinct chromatin states (Fig. 4G) best captured the human muscle hPTM landscape (see Materials and methods). State 1 and state 3 annotate genomic regions with high H3K18la in human muscle (Additional file 1: Fig. S8A-B). Both states are highly enriched for published human muscle enhancers (Fig. 4G). State 1 is also marked by H3K27ac but not by H3K4me3, encompasses mainly non-CGI-promoters, intronic regions, and 3-UTRs (Fig. 4G and Additional file 1: Fig. S8B) and it is only enriched for dELS and not for other cCREs. State 3 on the other hand is marked by all active marks, is enriched in CGI-promoters and exons (Fig. 4G and Additional file 1: Fig. S8A), and overlaps with PLS, pELS, and dELS. The other states are not marked by H3K18la, but represent active promoter regions (state 4, high in H3K27ac and H3K4me3; Additional file 1: Fig. S6C), repressed promoters (state 5, high H3K27me3), heterochromatin (state 7, high H3K9me3), or genic (state 2) and intergenic regions (state 6) devoid of any hPTMs profiled here. Like for the mouse samples, we note that H3K18la always co-localizes with H3K27ac, but that not all H3K27ac enriched regions are H3K18la enriched (e.g., state 4). Additionally, only ChromHMM states enriched in H3K18la overlap with published muscle enhancer annotations (states 1 and 3). Like for the mouse samples, we overlapped active hPTM peaks with putative enhancers (ENCODE’s cell-agnostic pELS and dELS). pELS were covered either by H3K4me3+H3K27ac+H3K18la, by H3K4me3+H3K27ac, or by H3K4me3 alone (Fig. 4H), in accordance with the mouse and promoter data. Many dELS were marked only by H3K18la, further endorsing the corresponding mouse data which suggested H3K18la to have a unique role in enhancers. Similarly, we found that human muscle H3K18la peaks were enriched more at cell type agnostic dELS than H3K27ac (see the “Materials and methods” section) (Fig. 4I). H3K18la overlapped with 51% of a published set of human muscle enhancers [57] (Fig. 4J). Notably, also H3K4me3 showed a strong overlap with the muscle enhancers (43%), which might be a consequence of how these enhancers were defined: presence of H3K27ac and H3K4me1, without any exclusion with regard to overlap with/vicinity to TSS [57], hence not excluding promoter regions.

Lastly, we correlated enhancer hPTM levels with (public) gene expression dataset, using the same strategy described above, i.e., linking each dELS to its closest but non-overlapping promoter. We found that H3K18la showed a positive, although overall weak, correlation between dELS hPTM levels and gene expression (*R* = 0.21), which was similar to H3K27ac (*R* = 0.20) (Additional file 1: Fig. S7A). Nevertheless, genes linked to the 2000 dELS with the highest H3K18la levels were enriched in muscle-specific GO terms (Fig. 4K). This was also true for H3K27ac-marked dELS but not for H3K4me3 (Additional file 1: Fig. S7B).

Overall, the human muscle data also showed a conserved role of H3K18la in marking tissue-specific active enhancers and active CGI promoters.

## Discussion

In agreement with prior studies, we found in all investigated tissues that H3K18la is present in a set of regions enriched for CGI promoters (Figs. 1D–E and 4B, C; ChromHMM state 8 in Fig. 2E) and many of these CGI promoters do belong to housekeeping genes (Fig. 2E, Additional files 4 and 5: Table S2-3) [45, 46]. Recent findings suggest that human housekeeping genes are primarily regulated by enhancer-like sequences contained within their promoter regions and not (or less) by distant enhancers [58]. An earlier report also showed that the majority of *Drosophila* housekeeping gene enhancers lie within 200 bp from a TSS, while developmental gene enhancers are predominantly found in intronic or intergenic regions [59]. This suggests that H3K18la in CGI-promoters may be primarily marking promoter-embedded enhancer-like sequences.

Globally, we found that the genomic distribution of H3K18la resembles H3K27ac (an established mark of active promoters and enhancers) better than H3K4me3 (Figs. 1B and 4A, Additional file 1: Fig. S6A). In promoter regions, H3K18la primarily marks promoters that are also marked by H3K27ac and H3K4me3, while the latter two also mark many promoters not marked by H3K18la (Figs. 1F and 4D). Genes with promoters marked by H3K18la (and H3K27ac and H3K4me3) are higher expressed than those without H3K18la (but with H3K27ac and H3K4me3) (Fig. 1H). Our analysis also showed that H3K18la distinctively marks active enhancers not overlapping with promoter regions (~dELS) (Fig. 2B, C), many of which are also marked by H3K27ac (Fig. 2A, D). Despite their overall genomic similarity, H3K27ac and H3K18la profiles also show clear distinctions: H3K27ac marks more promoters than H3K18la and H3K18la is found at more putative enhancers (dELS) than H3K27ac (Figs. 1F, H; 2A, D; and 4D, F-I, Additional file 1: Fig. S8A-C). Following the observation that H3K18la is enriched at active enhancers, we also found that H3K18la marks the majority of all public tissue-specific enhancers, corresponding to the samples included in this study, in a tissue-specific manner (Figs. 2C, E and 4G, J).

Given that there are no HiC datasets available for all tissues and conditions included in this manuscript, nor are the computational methods well enough established to define all enhancers in silico [60, 61], we cannot finally exclude that a specific fraction of tissue-specific enhancers is not marked by H3K18la. It is equally unlikely that the published enhancer sets are a perfect representation of the true set of active enhancers or that such a universal “true set” exists, as the set of active enhancers within one tissue type are known to change upon external environmental influences [57, 62]. Indeed, the many putative enhancers (cell-type-agnostic dELS) covered by H3K18la, and not by H3K27ac (Figs. 2D and 4H), suggest that H3K18la may have unique enhancer-related functions that differ from H3K27ac. Its specific role in promoters remains to be resolved.

On a side note, our PIM H3K18la profiles showed greater overlap with the published BMDM-specific enhancer set than our BMDM H3K18la and H3K27ac profiles (Fig. 2C). This matches the observation that the active enhancer landscape of macrophages is extremely well-adapted to their microenvironment [62] and as a consequence, published macrophage enhancer sets vary widely across studies [37, 44, 62]. It is likely that our PIM H3K18la profiles cover the defined enhancer regions in BMDMs from this particular study [37] better than the H3K18la and/or H3K27ac profiles of our BMDMs. Overall, there is still a considerable, tissue type-specific overlap between our H3K18la profiles and published ChIP-seq profiles (Additional file 1: Fig. S4A), and enhancer sets, both in absolute number of enhancers covered (Fig. 2C) as well as in tissue-specific H3K18la ChromHMM state enrichment over tissue-specific enhancers (Fig. 2E), which supports the validity of our findings.

The observation that H3K18la and H3K27ac profiles show feature-specific differences also raises the question whether both acylations are established by the same epigenetic machinery, including p300 [5], as has previously been proposed also for other histone acylation marks [63, 64]. Our results suggest that the differences in localization of H3K18la and H3K27ac are purposeful and as such regulated. This could be achieved through various mechanisms, such as specific histone lactylation writers (or erasers), regulation of the pool and concentration of lactyl-coA within the nucleus, being only available at specific genomic regions, or DNA-motif-specific or co-factor-based modulation of p300 activity, specificity, and recruitment. More in detail biochemical and genetic work is needed to answer these questions and reveal new insights into the organization and complexity of the histone code.

## Conclusion

We investigated the genomic distribution of H3K18la in human and mouse tissues, spanning a broad spectrum of differentiation states. Our supervised (overlap with public data) and unsupervised (ChromHMM) analysis revealed that H3K18la marks, in addition to active promoters, active tissue-specific enhancers. Provocatively, our analyses suggest that H3K18la at active CGI promoters may primarily mark promoter-embedded enhancer sequences, rendering H3K18la an enhancer-only marking hPTM with a partially distinct profile from H3K27ac.

## Materials and methods

### Cell culture

All cells were cultured in a humidified incubator at 37°C and 5% CO_2_.

#### BMDM

Bone marrow precursor cells were flushed out of the femur and tibiae bones with a syringe and needle and cultured for 7 days in DMEM, 20% heat-inactivated fetal bovine serum (FBS), 100 U/mL penicillin-streptomycin (P/S; Gibco, 15140122), and 40 ng/ml of recombinant M-CSF (PeproTech, 315-02). After 7 days, macrophages were collected, seeded in DMEM containing 10% heat-inactivated FBS, and 100 U/mL P/S for 24 h before harvesting.

#### ESC

Primed mouse ESC (mESC-ser) were cultured on 0.1% gelatin in DMEM supplemented with 15% FBS (Gibco), 2 mM GlutaMAX^TM^ (Gibco, 35050087), 0.05 mM β-mercaptoethanol (Gibco, 31350010), 100 U/mL P/S, 1X non-essential amino acids (Gibco, 11140035), and 10 ng/mL mLIF (Cambridge Stem Cell Institute). Naïve mESC (mESC-2i) were cultured in N2B27 supplemented with 1 μM MEK inhibitor (PD0325901; Cambridge Stem Cell Institute), 3 μM GSK3 inhibitor (CHIR99021; Cambridge Stem Cell Institute), and 10 ng/mL mLIF. The medium was changed daily. For lactate treatment, the cell medium was supplemented with 10 mM sodium-L-lactate dissolved in PBS.

#### MB and MT

Primary myoblast (MB) isolation was performed as described previously [65]. MBs were cultured in a growth medium containing a 1:1 ratio of DMEM (ThermoFisher Scientific, 12320032) and Ham’s F-10 (1×) nutrient mix (ThermoFisher Scientific, 22390058) supplemented with 10% horse serum (HS, ThermoFisher Scientific, 16050-122), 20% FBS, and 10 ng/ml basic-FGF (ThermoFisher Scientific, PHG0266). MB were cultured on dishes coated with Matrigel Basement Membrane Matrix (Corning, #356237, 1/25 dilution). When cells reached 80% confluency, the growth medium was switched to differentiation medium containing DMEM, 2% HS, and 100 U/mL P/S. MBs were fully differentiated into MTs after 3 days of differentiation. For lactate treatment, the cell medium was supplemented with 10 mM sodium-L-lactate dissolved in PBS. For WB and CUT&Tag, MBs and MTs were washed with PBS and harvested with trypsin.

### Lactate measurement

Intracellular and extracellular lactate concentration was measured using the Lactate Glo^TM^ Assay (Promega, J5022). Cells were seeded in a 96-well plate for lactate measurement. At the desired time point, media was collected for extracellular lactate concentration quantification. For intracellular lactate quantification, cells were washed twice with PBS before being lysed with 0.2 N HCl. Cell lysates were then neutralized with 1 M Tris-base before being incubated with the detection reagent. The luminescence was recorded with a CLARIOstar plate reader (BMG Labtech) after 1 h incubation. Extracellular lactate secretion was measured in the medium through background subtraction from fresh medium. Both intracellular and extracellular lactate concentration was determined from a standard curve. Lactate levels were normalized to total protein content (Qubit Protein Assay, Thermo Fisher Scientific, Q33211).

Naïve and primed mESC were seeded at 7500 cells/well and 5000 cells/well, respectively, and were grown in their respective media for 48 h before intracellular lactate concentration was measured. Extracellular lactate secretion was measured from 24-h incubation with fresh media. MB were seeded at a density of 7500 cells/well on a 96-well plate 5 days before the assay. Cellular differentiation into MT was initiated the following day by switching medium. Two days before the assay, fresh MB were plated at 4000 cells/well, concurrently to a medium change to the MT. One day before the assay, a medium change was performed to both MB and MT to ensure comparability with the other non-muscle cell types.

### Western blot

Histone extracts were prepared with the EpiQuik Total Histone Extraction kit (Epigentek, OP-0006-100-EP; for MB, MT, and GAS) or the acid histone extraction protocol published by Abcam (mESC, ADIPO, BMDM, and PIM). Histone protein extracts were resolved using a gradient SDS-PAGE before being immunoblotted onto a PVDF membrane. The membrane was blocked for 1 h in blocking solution (TBS/0.1% Tween/5% BSA or milk) and then incubated overnight at 4°C with primary antibodies diluted in blocking solution. After washes with TBS/0.1% Tween, the membranes were incubated with secondary antibodies conjugated with fluorescent or HRP tag diluted in blocking buffer for 1 h at room temperature. The band signals were visualized using Bio-Rad ChemiDoc Imaging System. The following primary antibodies were used: H3 (Abcam, ab1791), pan-KLA (PTM Bio, PTM-1401), and H3K18la (PTM Bio, PTM-1406 or PTM-1406RM). The secondary antibodies used were an HRP-conjugated monoclonal donkey anti-rabbit IgG (1:5000, Amersham, NA934) and StarBright Blue 700 Goat Anti-Rabbit IgG (1:2500, Bio-Rad, 12004161)

### Mouse biopsies

Female C57Bl6/J mice, aged 8–12 weeks, were housed in individually ventilated cages (3–4 littermates per cage) in standard housing conditions (22°C, 12 h light/dark cycle), with ad libitum access to chow diet and water. Health status of all mice was regularly monitored according to FELASA guidelines. Muscle and macrophage samples were collected form mice which were anesthetized using Ketamine (80–100 mg/kg) and Xylazine (10–15 mg/kg) via intraperitoneal injection 5 min before sacrifice. M. gastrocnemius (GAS) samples were harvested and snap-frozen in liquid nitrogen. For adipocyte samples, epididymal adipose tissues (ADIPO) from euthanized (CO_2_) 10-week-old female AdipoCre-NuTRAP [66] mice were extracted and snap-frozen in liquid nitrogen.

### Isolation of post-ischemia macrophages from muscle

Hind-limb ischemia experiments were performed as described before with minor modifications [67, 68]. Briefly, mice were anesthetized with isoflurane. The hind limb was shaved, and the skin was incised. The proximal end of the femoral artery and the distal portion of the saphenous artery were ligated. The artery and all side-branches were dissected free the femoral artery and attached side-branches were excised. Ischemia induces muscle damage due to hypoxia and consequently macrophage recruitment. Two days after the onset of hindlimb ischemia, the calf muscle from the ischemic limb was collected and digested in 2 mg/ml Collagenase IV (Thermo Fisher Scientific, 17104019) / Dispase II (Sigma-Aldrich, D4693-1G) for 1 h at 37°C. After filtration and washing steps, red blood cells were removed with ACK Lysis buffer (Gibco, A1049201). Then, CD45^+^CD11b^+^F4/80^+^CD64^+^ macrophages were stained and sorted (Sony Cell sorter SH800S) for either histone isolation or CUT&Tag. The used antibodies were the following: PE anti-mouse CD45 (Biolegend [30-F11], 103106), PerCP/Cy5.5 anti-mouse/human CD11b (Biolegend [M1/70], 101228), Alexa Fluor® 488 anti-F4/80 Rat Monoclonal Antibody (Biolegend [clone: BM8], 123120), APC anti-CD64 Mouse Monoclonal Antibody (Biolegend [clone: X54-5/7.1], 139306).

### Human biopsies

Samples were obtained from the ACTIBATE study [69]. Only control samples from female participants were included here. The ACTIBATE study is an RCT, registered at ClinicalTrials.gov (ID: NCT02365129). The Human Research Ethics Committee of both University of Granada (n° 924) and Servicio Andaluz de Salud (Centro de Granada, CEI-Granada) approved the study design, study protocols, and informed consent procedure. All participants have provided written informed consent. The study was performed following the ethical guidelines of the Declaration of Helsinki, last modified in 2013. The biopsies were collected using the Bergstrom technique by an expert surgeon.

### Nuclei isolation

All buffers were supplemented with 5 mM sodium-butyrate (Sigma, 303410) and 1X complete protease inhibitor (Merck, 11873580001).

#### BMDM, PIM, MB, and MT

Sorted macrophages, MB, or MT samples were centrifuged for 5 min at 4°C, 500 rpm; the supernatant was removed; and the cells were lysed on ice in 1 mL of nucleus extraction buffer (1× prelysis buffer from the EpiGentek EpiQuick Total Histone Extraction Kit, OP-0006-100). To stop the lysis reaction, 1 mL of PBS+1%BSA was added, and nuclei were collected through centrifugation for 5 min at 4°C, 500 rpm. The supernatant was removed, the nuclei were resuspended in PBS+1% BSA, and a sample was visually inspected for viability, purity, and abundance of nuclei under the microscope.

#### Muscle samples (mouse and human)

Muscle samples were thawed and sliced in small pieces on ice. Subsequently, the pieces were transferred to an ice-cold dounce homogenizer (7 mL) and 3 mL of nucleus extraction buffer (1× prelysis buffer from the EpiGentek EpiQuick Total Histone Extraction Kit, OP-0006-100) was added before douncing, on ice, 10× with pestle A and 10× with pestle B. To stop the lysis reaction, 3 mL of PBS+1%BSA was added. After 5-min centrifugation at 4°C, 500 rpm, the supernatant was removed, the nuclei were resuspended in PBS+1%BSA, and a sample was visually inspected for viability, purity, and abundance of nuclei under the microscope.

#### Adipocytes

Nuclei were isolated using a Kimble 7-ml glass douncer using ice-cold nuclei isolation buffer (10 mM Tris-HCl pH 7.4, 3 mM MgCl_2_, 10 mM NaCl, 0.1 % Igepal-CA630, 1x protease inhibitor) and washed two times with PBS-BSA 1%. M-280 Streptavidin Dynabeads™ (ThermoFisher, 11205D) were washed two times with PBS-BSA, and nuclei were bound to beads, while rotating at 4°C for 30 min. After binding, beads were washed 3 times with PBS-BSA.

### CUT&Tag

CUT&Tag was performed according to the published CUT&Tag protocol [29] for nuclei (BMDM, PIM, MB, MT, GAS, ADIPO) or cells (ESC). All buffers were supplemented with 5 mM sodium-butyrate (Sigma, 303410) and 1X complete protease inhibitor (Merck, 1187358000). Protein lo-bind tubes (Eppendorf, EP0030108116) were used to reduce sample loss. For GAS samples, incubation volumes were doubled to account for the tissue debris that remained in the nuclear suspensions since this gave better Tapestation QC results. Antibodies against H3K18la (PTM-Bio, PTM-1406), H3K4me3 (Abcam, ab8580), H3K27me3 (Cell Signaling Technology, C36B11), H3K27ac (Abcam, ab4729), and H3K9me3 (Abcam, ab8898) were used in this study. Libraries were indexed using Nextera Indexes, and 150-bp paired-end sequencing was performed on Illumina Novaseq instruments.

### RNA library preparation and sequencing

#### ESC

Total RNA for each sample was extracted using RNeasy mini kit (QIAGEN, 74104). Extracted RNA was PolyA-enriched. Then, RNA was used for library preparation using the TruSeq RNA Library Prep Kit v2 (Illumina) following the manufacturer’s instructions. Libraries were indexed using Illumina Indexes and 50 bp single-end sequencing was performed on Illumina HiSeq 2000 instruments.

#### Muscle samples

To each GAS sample, 1 stainless steel bead together with 1ml of ice-cold TRIzol (ThermoFisher Scientific, 15596018) was added. The mixtures were homogenized for 7 min using a bead mill at 50 rpm (Qiagen TissueLyzer LT, 85600). The beads were removed using forceps cleaned with RNAzap (Thermo Fisher Scientific, AM9780/AM9782) after each transfer to avoid carryover of RNA. The addition of 200 μl of chloroform (VWR, 22711.324) was followed by vigorous shaking for 15–20 s and a centrifugation step of 15 min at 14,000 rcf at 4°C. The aqueous phase was transferred into a new vial and mixed with an equal amount of 100% ethanol. RNA was then extracted using the RNA Clean & Concentrator^TM^-25 Kit (Zymo Research, R1017 & R1018). The whole volume was transferred to a Zymo-Spin^TM^ IICR-column in a collection tube, spun down for 30 s at 10,000 rpm, and the flow-through discarded. Next, a mixture of 5 μl DNAse and 75 μl DNA digestion buffer was added to the column and incubated for 15 min at room temperature. Four hundred microliters of RNA Prep buffer was added directly on top of the DNase solution and the samples were spun down for 30 s (4°C, 10000 rpm). This process was repeated twice from the addition of 700 μl of RNA Wash buffer, spinning down for 30 s at 4°C (10,000 rpm) and removal of flow through, followed by the addition of 400 μl of RNA wash buffer and a 2-min centrifugation step with the same settings. After the final removal of flow through, 30 μl of DNase/RNase-Free Water was added directly onto the column matrix. The column was placed in a fresh vial and once again spun down for 30 s (4°C, 1,0000 rpm). PolyA enriched mRNA sequencing libraries were prepared by Novogene, UK, and 150-bp paired-end sequencing was performed on Illumina Novaseq instruments.

#### MB and MT

Total RNA from myoblasts and myotubes was isolated using TRIzol extraction (ThermoFisher Scientific, 15596018). Purification of extracted RNA was performed using Zymo RNA Clean and Concentrator Kit with DNase (Zymo Research, R1018) according to the manufacturers’ instructions. Quantification of total RNA was performed using a nanodrop system. Five nanograms of RNA was used as an input for the Smart-seq2 protocol as described in Picelli et al. [70, 71] including cDNA synthesis, pre-amplification, tagmentation, and enrichment steps.

### Data processing

All genomic data were processed using pipelines built in Nextflow [72] v21.04.3, adapted from the Babraham Institute GitHub repository (https://github.com/s-andrews/nextflow_pipelines) for reproducible data analysis.

#### CUT&Tag

Quality control of the raw sequencing reads was performed using FastQC [73] v0.11.9. Raw reads were trimmed off low-quality bases and adapter sequences using TrimGalore v0.6.6 (https://github.com/FelixKrueger/TrimGalore). Filtered reads were aligned against the reference mouse genome assembly mm10 in case of mouse samples and human genome assembly GRCh38 in case of human samples using Bowtie2 [74] v2.4.4 with options: *--end-to-end --very-sensitive --no-mixed --no-discordant --phred33 -I 10 -X 700*. Aligned bam files were sorted based on chromosomal coordinates using the *sort* function of samtools [75] v1.13. Sorted bam files were summarized into bedgraph files using the *genomecov* function of bedtools v2.30 (Quinlan et al, 2010). In case of samples with multiple biological replicates, replicate specific bedgraph files were combined using the *unionbedg* function of bedtools [76] v2.30. Peak calling was performed on all bedgraph files using SEACR [30] v1.3 in stringent mode by selecting the top 1% of called peaks. SEACR is specifically developed for CUT&RUN and is likewise the recommended pipeline for chromatin profiling data with very low background like CUT&Tag. Visual QC of bam files and called peaks were performed using Seqmonk [77].

#### RNA-seq

Quality control of the raw sequencing reads was performed using FastQC [73] v0.11.9. Raw reads were trimmed off low-quality bases and adapter sequences using TrimGalore v0.6.6 (https://github.com/FelixKrueger/TrimGalore). Filtered reads were aligned against the reference mouse genome assembly mm10 in case of mouse samples and human genome assembly GRCh38 in case of human samples using HISAT2 [78] v2.2.1. Raw gene counts were quantified using the *featureCounts* program of subread [79] v2.0.1.

### Data analysis

#### CUT&Tag sample clustering

For the binned clustering analysis, the genome was split into bins of 3000 bp and for each bin, in each sample, reads were counted using the *summarizeOverlaps* function from the R package GenomicsAlignments v1.8.4. For each sample, the per-bin read count was normalized to the total number of mapped reads, log2 normalized, and used as input for the *plotMDS* function (see further in the “Data visualization” section).

#### CUT&Tag peaks

For samples with multiple biological replicates, only peaks called on merged bedgraph files were considered for downstream analysis. Peaks overlapping with mouse and human blacklist regions [80] were filtered out.

Called peaks for each sample were combined to create a master (union) peak list (https://yezhengstat.github.io/CUTTag_tutorial/). This master peak list was used as a reference to generate the fragment count matrix of all samples using the R package chromVAR [81] v1.16.

Called peaks were annotated with the R package ChIPseeker [82] v1.30.3 [83]. Peak fold enrichment values were calculated using the formula: Σ (bp overlap) * genome_size /[Σ (bp PTM peak)*Σ (bp genomic feature)]. Different genomic features including CpG island tracks were downloaded using the R package annotatr [73] v1.20. Promoter regions were defined as 2000 bp up- and downstream of TSS.

#### CUT&Tag peak overlaps with promoters / enhancers

Peaks overlapping with promoters were extracted using the annotatePeak function from the R package clusterProfiler v4.0.5 ChIPseeker [82] v1.30.3, selecting only the peaks with promoter annotation for further analysis. The promoter regions were defined using the getPromoters function from the R package ChIPseeker [82] v1.30.3, using the TxDb.Mmusculus.UCSC.mm10.knownGene database as input, setting the TssRegion to c(–2000, 2000). For the pELS/dELS overlap, peaks were overlapped with cCRE using the bedtools [76] function intersect. The groups of genes with different hPTM promoter/dELS/pELS occupation combinations were calculated using the venn function from the R package gplots v3.1.1.

For the cistrome transcription-factor binding analysis, the promoter regions of the genes covered by different hPTM combinations were used as input to the online Cistrome database analysis tool [31] using the settings “All peaks in each sample” and “Transcription factor, chromatin regulator”.

#### ChromHMM analysis

Tissue-specific H3K18la chromatin states were identified using the ChromHMM [33] v1.22 software. The bam files of all mouse H3K18la samples were binarized into default 200 bp bins using the function *BinarizeBam*. Models with different number of chromatin states starting from 1 to 20 were learned from the binarized data using the function *LearnModel*. All twenty models were compared against each state of the reference model, i.e., the model with maximum number of states based on the emission parameter correlation using the function *CompareModel*. Once the model was finalized with defined number of chromatin states, the state fold enrichment was performed against genomic features, tissue-specific enhancers, ENCODE’s cCREs, public ChIP-seq data sets, and house-keeping genes [38] and their gene promoters using the function *OverlapEnrichment* [38, 84]. For mouse mESC-ser, GAS, PIM samples, and human muscle samples, chromatin states were identified in the same way using the available hPTMs (H3K18la, H3K4me3, H3K27ac, H3K27me3 for mouse samples; H3K18la, H3K4me3, H3K27ac, H3K27me3, H3K9me3 for human samples).

#### CUT&Tag peak/RNA-seq correlation

Tissue-specific fragment count matrices were generated by quantifying the reads present in promoter/dELS regions using the R package chromVAR [81] v1.16. Raw CUT&Tag fragment count matrices and RNAseq gene count matrices were normalized into CPM and RPKM (gene length correction) values respectively using the R package edgeR [85] v3.28. Normalized fragment counts were summarized at the gene level.

For each tissue, an integrated data set was created linking gene expression to hPTM levels in the corresponding promoter or dELS region. Genes closest to dELS were found using bedtools [76] *closest* function. For tissue-matching hPTMs and RNAseq samples, the normalized counts are averaged over biological replicates, if available. For each tissue, highest and lowest expressed genes were defined based on their average log normalized RPKM values.

#### Differential H3K18la peak/gene expression correlation analysis

For each pair of samples (MT and MB; ESC-ser and ESC-2i), H3K18la peaks were combined to generate a cell type pair-specific master (union) peak list. These master peak lists were used to generate the cell type pair-specific fragment count matrices using the R package chromVAR [81] v1.16. Each fragment matrix was subset by promoter or dELS regions using the function *findOverlaps* of the R package GenomicRanges.

#### Differential gene expression analysis after lactate treatment

EdgeR was used to identify differentially expressed genes using nominal *P* < 0.01 and abs(log2FC) > 0.5 as thresholds.

#### Public datasets

##### ChIP-seq

Data from the following studies were obtained to compare our results. Peaks were directly downloaded and used as such from the publications’ supplemental data. Mouse gastrocnemius peaks were obtained from Rovito et al*.* [35]. mESC peaks were obtained from Perino et al. [40] and ENCODE [34]. Mouse BMDM peaks were obtained from Zhang et al. [5] and ENCODE [34]. Mouse MB and MT peaks were obtained from Asp et al. [39].

##### RNA-seq

PIM RNA-seq data was obtained from Zhang et al. [27]. Human muscle RNA-seq data was obtained from Williams et al*.* [57]. Public RNAseq data were re-processed using our in-house pipeline to obtain comparable raw count matrices as mentioned above (see the “Data processing”/“RNA-seq” sections).

##### Enhancer sets

mESC enhancers were obtained from the SCREEN project from ENCODE, using pELS and dELS regions from the E14-specific cCRE-set [34]. Mouse muscle (gastrocnemius) enhancers were obtained from Rovito et al. [35]. Mouse macrophage enhancers were obtained from Denisenko et al. [37]. Mouse myoblast and myotube enhancers were obtained from Blum et al*.* [36]. Human muscle enhancers were obtained from Williams et al. [57]. Overlap between different genomic regions/peak sets was obtained using bedtools [76] *intersect* function. Intersects from 1 bp of intersection were included in downstream analysis. Enrichment was calculated as Σ (bp overlap)/[Σ (bp set1)*Σ (bp set2)].

#### Functional enrichment analysis

Gene ontology enrichment analysis was performed using the function *enrichGO* from the R package clusterProfiler [84] v.4.0.5, using the Benjamini-Hochberg *p-*value adjustment method, searching for all ontology categories, using the 3.13.0 versions of org.Mm.eg.db [86] and org.Hs.eg.db [87]. Comparative GO analysis was performed using the *compareCluster* function from the R package clusterProfiler [84] v.4.0.5 using the same settings.

### Data visualization

Multidimensional scaling (MDS) plots were generated using the *plotMDS* function in the R package limma v.3.48.3. All heat maps were generated using the R package pheatmap [88] v1.0.12. Correlation scatter plots were made using the *ggscatter* function from R package ggpubr v0.4. CUT&Tag peak distribution across different genomic features and peak profiles around TSS were visualized using the functions *plotAnnoBar*, and *plotDistToTSS* from R package ChIPseeker [82] v1.30.3. Venn diagrams were created using the *ggVennDiagram* function from the ggVennDiagram [89] R package v.1.1.4. GO analysis results were visualized using the *dotplot* function of the R package enrichplot v.1.12.2. The *boxplot* function from the R package Graphics [90] was used to plot boxplots.

### Statistics

All statistical and other data analyses mentioned above were performed using the statistical programming language R [91] v4.1.0 or above. For correlation analyses, Pearson correlation tests were performed for hPTM versus hPTM and Spearman correlation coefficients were used for hPTM vs gene expression. Group values were compared using two-sided Mann-Whitney *U* tests. Statistical significance was called from (adjusted) *p* < 0.05.

## Supplementary Information

Additional file 1: Supplemental figures. Fig S1, Fig S2, Fig S3, Fig S4, Fig S5, Fig S6, Fig S7, Fig S8.

Additional file 2: Uncropped western blot images. Cropped images used in Fig. 1A and Fig S1B.

Additional file 3: Table S1: Quality Control metrics. For each sample included in this study, the following data is provided: tissue of origin, hPTM profiled, biological replicate, reads (million), GC content (%), aligned fraction of reads (%), number of called peaks.

Additional file 4: Table S2: Genes whose promoters lie in state 8 from the mouse H3K18la ChromHMM. Data used in Fig. 2E.

Additional file 5: Table S3: GO categories of genes whose promoters lie in state 8 from the mouse H3K18la ChromHMM. Data used in Fig. 2E. The following data is provided: GO ontology category, GO identifier number, GO term description, GO gene ratio, GO background ratio, p-value, adjusted p-value, q-value, gene entrez ids, gene count.

Additional file 6: Table S4: Genes expression changes in MB treated with 10 mM lactate. Data used in Fig. 3H. edgeR outcome from differential expression test of control MBs versus MBs treated with 10 mM sodium-L-lactate (see ‘Materials and methods’). The following data is provided: ensembl gene id, gene entrez id, gene name, logFC, logCPM, p-value, FDR, regulation (up/down/non-significant), and whether the gene has a H3K18la-peak in its promoter region in MTs or MBs.

Additional file 7. Review history.

## Data Availability

*Deposition of sequencing data* Gene expression (RNA-seq) and all hPTM genomic profiling (CUT&Tag) datasets are available in GEO under the accession number GSE195860. Individual datasets are available under GSE195859 (MB, MT, and GAS RNA-seq [94]), GSE195856 (mouse CUT&Tag [95]), and GSE195854 (human CUT&Tag [96]). mESC RNAseq datasets are available under GSE196084 [97]. *Public sequencing data* Peaks obtained from ChIP-seq data were directly downloaded and used as such from the publications’ supplemental data. Mouse gastrocnemius peaks were obtained from Rovito et al. [35], which were derived from GSE142518 [98]. mESC peaks were obtained from Perino et al. [40], derived from GSE94300 [99], and ENCODE [34]. Mouse BMDM peaks were obtained from Zhang et al. [5], derived from GSE115354 [100], and ENCODE [34]. Mouse MB and MT peaks were obtained from Asp et al. [39] and derived from GSE25308 [101]. PIM RNA-seq data was obtained from GSE148584 [102], as published in Zhang et al. [27]. Human muscle RNA-seq data was obtained from GSE144134 [103], as published in Williams et al. [57]. Public RNAseq data were re-processed using our in-house pipeline to obtain comparable raw count matrices as mentioned above (see “Data processing”/“RNA-seq” sections).
