# Supplementary material for: H3K18 lactylation marks tissue-specific active enhancers
Source: Genome Biol. 2022 Oct 3;23:207. doi: 10.1186/s13059-022-02775-y (PMC9531456; doi:10.1186/s13059-022-02775-y)
Supplement: Supplementary file 1 — Additional file 1: Supplemental figures. Fig S1, Fig S2, Fig S3, Fig S4, Fig S5, Fig S6, Fig S7, Fig S8. [file 13059_2022_2775_MOESM1_ESM.pdf]

Fig S1

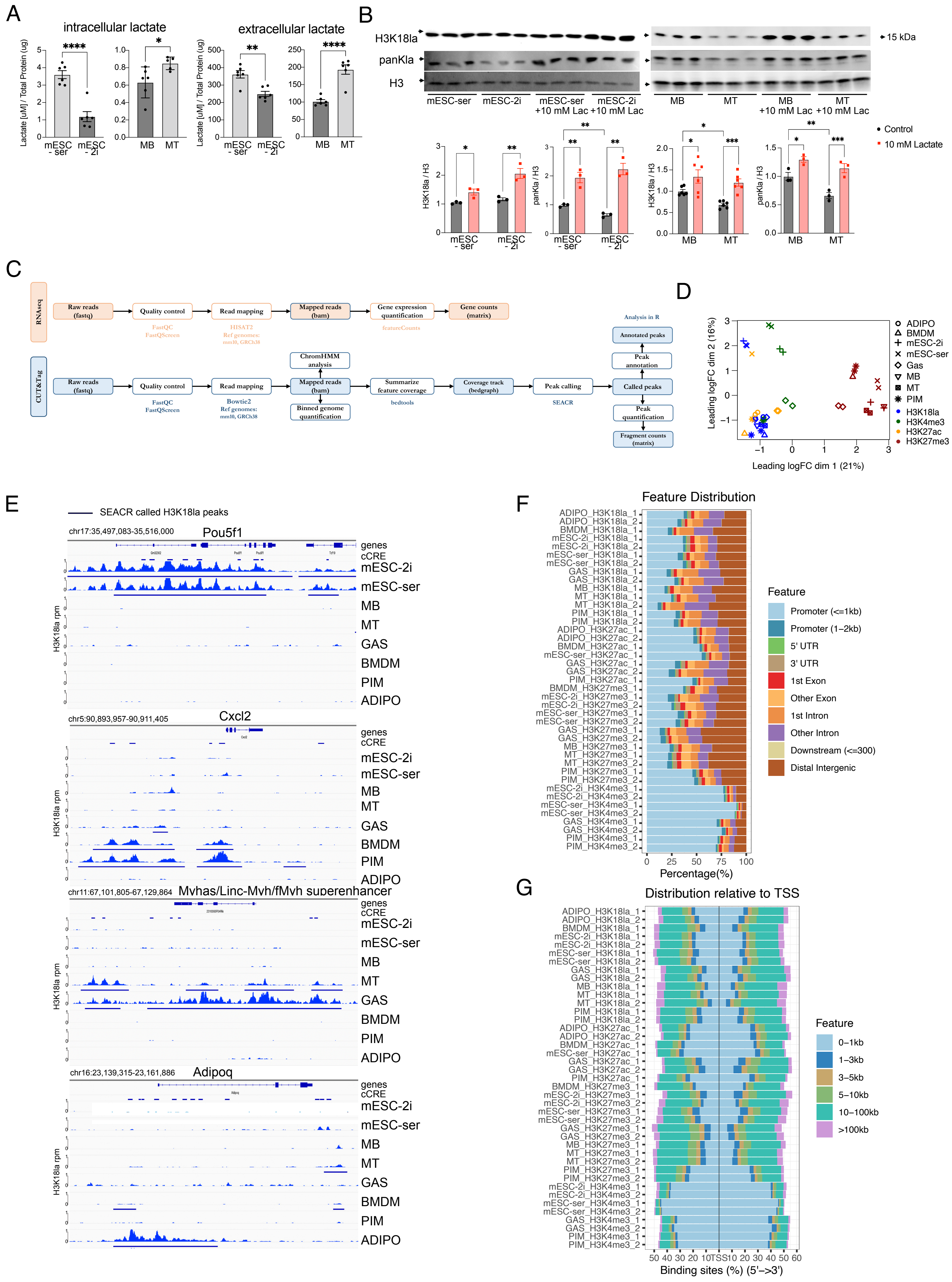
A
intracellular lactate
extracellular lactate
Lactate [uM] / Total Protein (ug)
mESC - ser
mESC - 2i
MB
MT
****
*
**
****
B
H3K18la
panKla
H3
15 kDa
mESC-ser
mESC-2i
mESC-ser +10 mM Lac
mESC-2i +10 mM Lac
MB
MT
MB +10 mM Lac
MT +10 mM Lac
H3K18la / H3
panKla / H3
Control
10 mM Lactate
C
RNAseq
Raw reads (fastq)
Quality control
Read mapping
Mapped reads (bam)
Gene expression quantification
Gene counts (matrix)
FastQC
FastQScreen
HISAT2
Ref genomes: mm10, GRCh38
featureCounts
CUT&Tag
ChromHMM analysis
Summarize feature coverage
Coverage track (bedgraph)
Peak calling
Binned genome quantification
Bowtie2
Ref genomes: mm10, GRCh38
bedtools
SEACR
Analysis in R
Annotated peaks
Peak annotation
Called peaks
Peak quantification
Fragment counts (matrix)
D
Leading logFC dim 1 (21%)
Leading logFC dim 2 (16%)
ADIPO
BMDM
mESC-2i
mESC-ser
Gas
MB
MT
PIM
H3K18la
H3K4me3
H3K27ac
H3K27me3
E
SEACR called H3K18la peaks
chr17:35,497,083-35,516,000
Pou5f1
chr5:90,893,957-90,911,405
Cxcl2
chr11:67,101,805-67,129,864
Myhas/Linc-Myh/fMyh superenhancer
chr16:23,139,315-23,161,886
Adipoq
genes
cCRE
mESC-2i
mESC-ser
MB
MT
GAS
BMDM
PIM
ADIPO
H3K18la rpm
F
Feature Distribution
Percentage(%)
Feature
Promoter (<=1kb)
Promoter (1–2kb)
5' UTR
3' UTR
1st Exon
Other Exon
1st Intron
Other Intron
Downstream (<=300)
Distal Intergenic
G
Distribution relative to TSS
Binding sites (%) (5'–>3')
Feature
0–1kb
1–3kb
3–5kb
5–10kb
10–100kb
>100kb

Additional File 1: Fig. S1: H3K18la is enriched at active promoters.

A) Intracellular and extracellular (secreted) lactate levels in mESC-2i, mESC-ser, MB and MT. Significant differences were calculated using Wilcoxon test. B) Western blots of H3K18la, panKla and H3 in mESC-2i, mESC-ser, MB and MT with or without supplemented sodium-L-lactate (10mM). Corresponding quantified relative expression of H3K18la and panKla to H3 are depicted as barplots. All mESC samples are normalized to control mESC-ser, all MB-MT samples are normalized to control MB. Significant differences were calculated using Wilcoxon test. C) Bioinformatics workflow illustrating the RNAseq and CUT&Tag data processing steps used in this manuscript. D) MDS of hPTMs profiled from various mouse samples, quantified over 3000 bp genome-wide tiles. E) IGV genome browser (1) snapshots of H3K18la profiles from various mouse samples and corresponding SEACR-called peaks. H3K18la levels are depicted (rpm). Genomic regions are indicated on the top, as well as RefSeq gene names. The selected regions include the gene *Pou5f1*, the gene *Cxcl2* and its flanks, the fast *Myh* super enhancer region (2), overlapping with *Linc-Myh* and *Myhas*, and the gene *Adipoq*. F) Distribution of hPTM peaks from all biological replicates across genomic features. G) Distribution of hPTM peak distances to TSS for all biological replicates. **p* value < 0.05, ***p* value < 0.01, ****p* value < 0.001, *****p* value < 0.0001.

1. Robinson JT, Thorvaldsdóttir H, Winckler W, Guttman M, Lander ES, Getz G, et al. Integrative genomics viewer. Nat Biotechnol. 2011 Jan;29(1):24–6.

2. Dos Santos M, Backer S, Auradé F, Wong MMK, Wurmser M, Pierre R, et al. A fast Myosin super enhancer dictates muscle fiber phenotype through competitive interactions with Myosin genes. Nat Commun. 2022 Dec;13(1):1039.

Fig S2

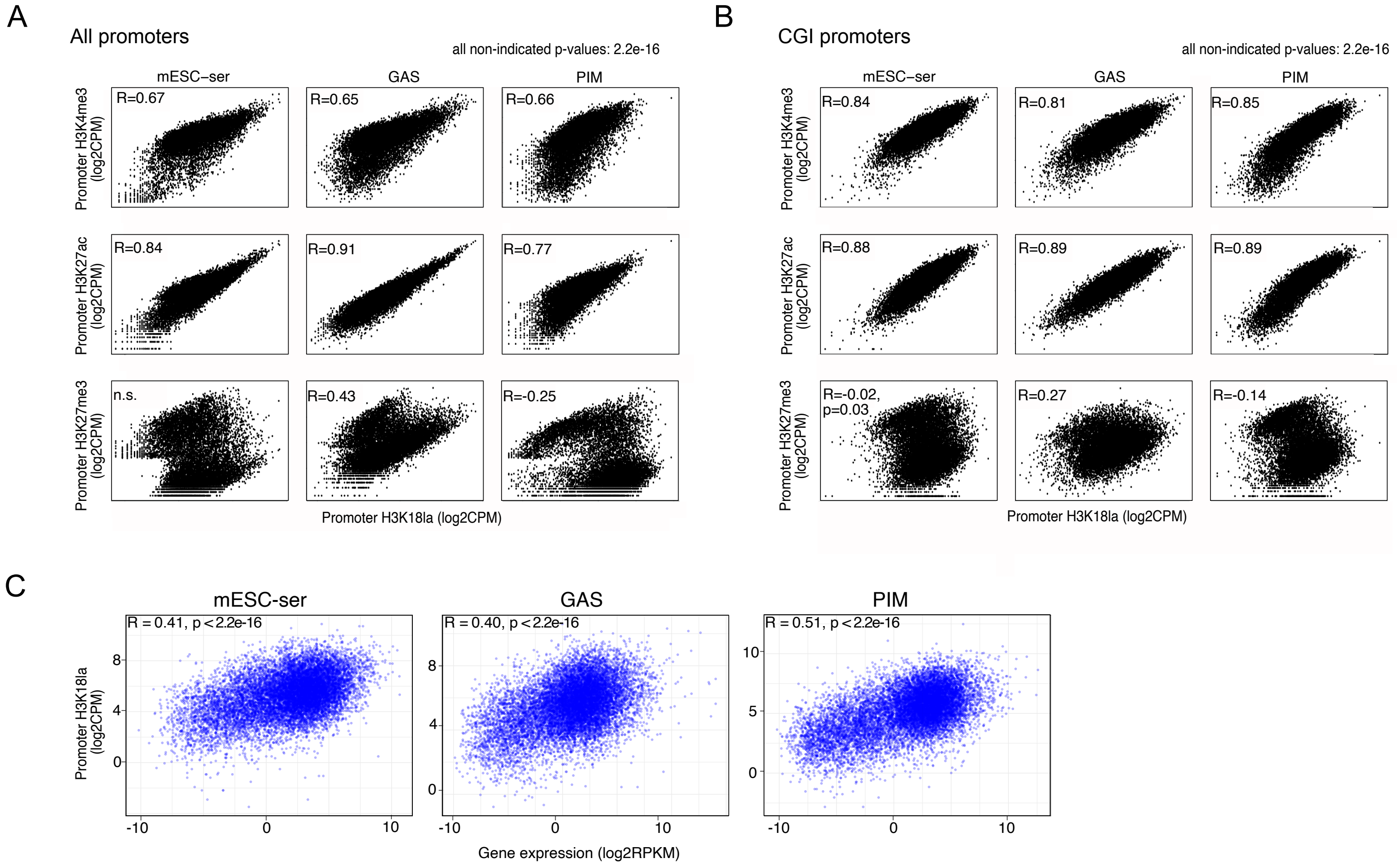
A
All promoters
all non-indicated p-values: 2.2e-16
mESC–ser
GAS
PIM
Promoter H3K4me3 (log2CPM)
R=0.67
R=0.65
R=0.66
Promoter H3K27ac (log2CPM)
R=0.84
R=0.91
R=0.77
Promoter H3K27me3 (log2CPM)
n.s.
R=0.43
R=-0.25
Promoter H3K18la (log2CPM)
B
CGI promoters
all non-indicated p-values: 2.2e-16
mESC–ser
GAS
PIM
Promoter H3K4me3 (log2CPM)
R=0.84
R=0.81
R=0.85
Promoter H3K27ac (log2CPM)
R=0.88
R=0.89
R=0.89
Promoter H3K27me3 (log2CPM)
R=-0.02, p=0.03
R=0.27
R=-0.14
Promoter H3K18la (log2CPM)
C
mESC-ser
GAS
PIM
R = 0.41, p < 2.2e-16
R = 0.40, p < 2.2e-16
R = 0.51, p < 2.2e-16
Promoter H3K18la (log2CPM)
Gene expression (log2RPKM)

Additional File 1: Fig. S2: Promoter H3K18la correlates to other active marks and gene expression.

A) Scatterplots showing the pairwise correlation between the H3K18la levels and other hPTM levels (log_2CPM) at all promoters for mESC-ser, GAS and PIM. Pearson's correlation coefficient R and p-values are indicated. B) Scatterplots showing the pairwise correlation between the H3K18la levels and other hPTM levels (log_2CPM) at CGI promoters for mESC-ser, GAS and PIM. Pearson's correlation coefficient R and p-values are indicated C) Scatter plots showing the correlation between CGI promoter H3K18la levels (log_2CPM, y-axis) and expression of the corresponding gene (log_2RPKM, x-axis) for mESC-ser, GAS and PIM. Spearman's correlation coefficient R and p-values are indicated.

Fig S3

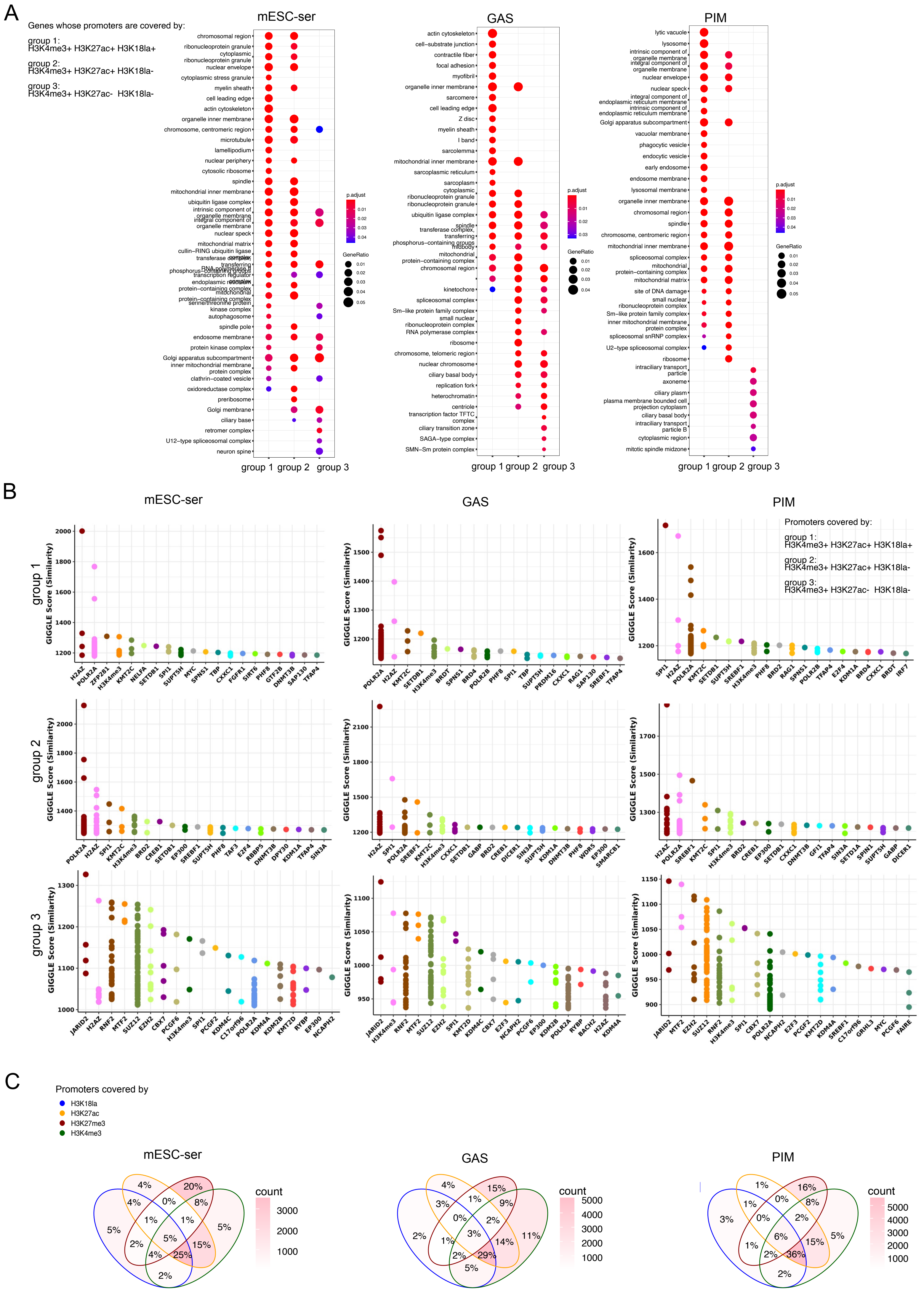

A
Genes whose promoters are covered by:
group 1:
H3K4me3+ H3K27ac+ H3K18la+
group 2:
H3K4me3+ H3K27ac+ H3K18la-
group 3:
H3K4me3+ H3K27ac- H3K18la-
mESC-ser
chromosomal region
ribonucleoprotein granule
cytoplasmic ribonucleoprotein granule
nuclear envelope
cytoplasmic stress granule
myelin sheath
cell leading edge
actin cytoskeleton
organelle inner membrane
chromosome, centromeric region
microtubule
lamellipodium
nuclear periphery
cytosolic ribosome
spindle
mitochondrial inner membrane
ubiquitin ligase complex
nuclear speck
mitochondrial matrix
autophagosome
spindle pole
endosome membrane
protein kinase complex
Golgi apparatus subcompartment
inner mitochondrial membrane protein complex
clathrin−coated vesicle
oxidoreductase complex
preribosome
Golgi membrane
ciliary base
retromer complex
U12−type spliceosomal complex
neuron spine
group 1 group 2 group 3
p.adjust
GeneRatio
GAS
actin cytoskeleton
cell−substrate junction
contractile fiber
focal adhesion
myofibril
organelle inner membrane
sarcomere
cell leading edge
Z disc
myelin sheath
I band
sarcolemma
mitochondrial inner membrane
sarcoplasmic reticulum
sarcoplasm
ribonucleoprotein granule
ubiquitin ligase complex
chromosomal region
kinetochore
spliceosomal complex
Sm−like protein family complex
RNA polymerase complex
ribosome
chromosome, telomeric region
nuclear chromosome
ciliary basal body
replication fork
heterochromatin
centriole
transcription factor TFTC complex
ciliary transition zone
SAGA−type complex
SMN−Sm protein complex
group 1 group 2 group 3
PIM
lytic vacuole
lysosome
nuclear envelope
nuclear speck
Golgi apparatus subcompartment
vacuolar membrane
phagocytic vesicle
endocytic vesicle
early endosome
endosome membrane
lysosomal membrane
organelle inner membrane
chromosomal region
spindle
chromosome, centromeric region
mitochondrial inner membrane
spliceosomal complex
mitochondrial matrix
site of DNA damage
Sm−like protein family complex
spliceosomal snRNP complex
U2−type spliceosomal complex
ribosome
axoneme
ciliary plasm
ciliary basal body
cytoplasmic region
mitotic spindle midzone
group 1 group 2 group 3
B
mESC-ser
GAS
PIM
Promoters covered by:
group 1:
H3K4me3+ H3K27ac+ H3K18la+
group 2:
H3K4me3+ H3K27ac+ H3K18la-
group 3:
H3K4me3+ H3K27ac- H3K18la-
group 1
group 2
group 3
GIGGLE Score (Similarity)
C
Promoters covered by
H3K18la
H3K27ac
H3K27me3
H3K4me3
mESC-ser
GAS
PIM
count

Additional File 1: Fig. S3: H3K18la marks active promoters important to tissue biology

A) Comparative GO enrichment analysis (category 'Cellular Component') for genes in group 1 (promoter occupied by H3K4me3+ H3K27ac+ H3K18la), group 2 (promoter occupied by H3K4me3+H3K27ac and not H3K18la) and group 3 (promoter occupied by H3K4me3 only and not by H3K27ac nor H3K18la) as defined in Figure 1F. B) Cistrome analysis of promoter regions of genes belonging to group 1, group2, or group 3. The similarity to the public transcription factor binding site datasets is indicated on the y-axis using the GIGGLE score. C) Venn diagrams depicting the promoter overlaps marked by various hPTMs in mESC-ser, GAS and PIM samples. Overlaps are coloured according to the absolute number of promoters marked by various combinations of hPTMs. Percentages indicate the fraction of actively marked promoters belonging to each group.

Fig S4

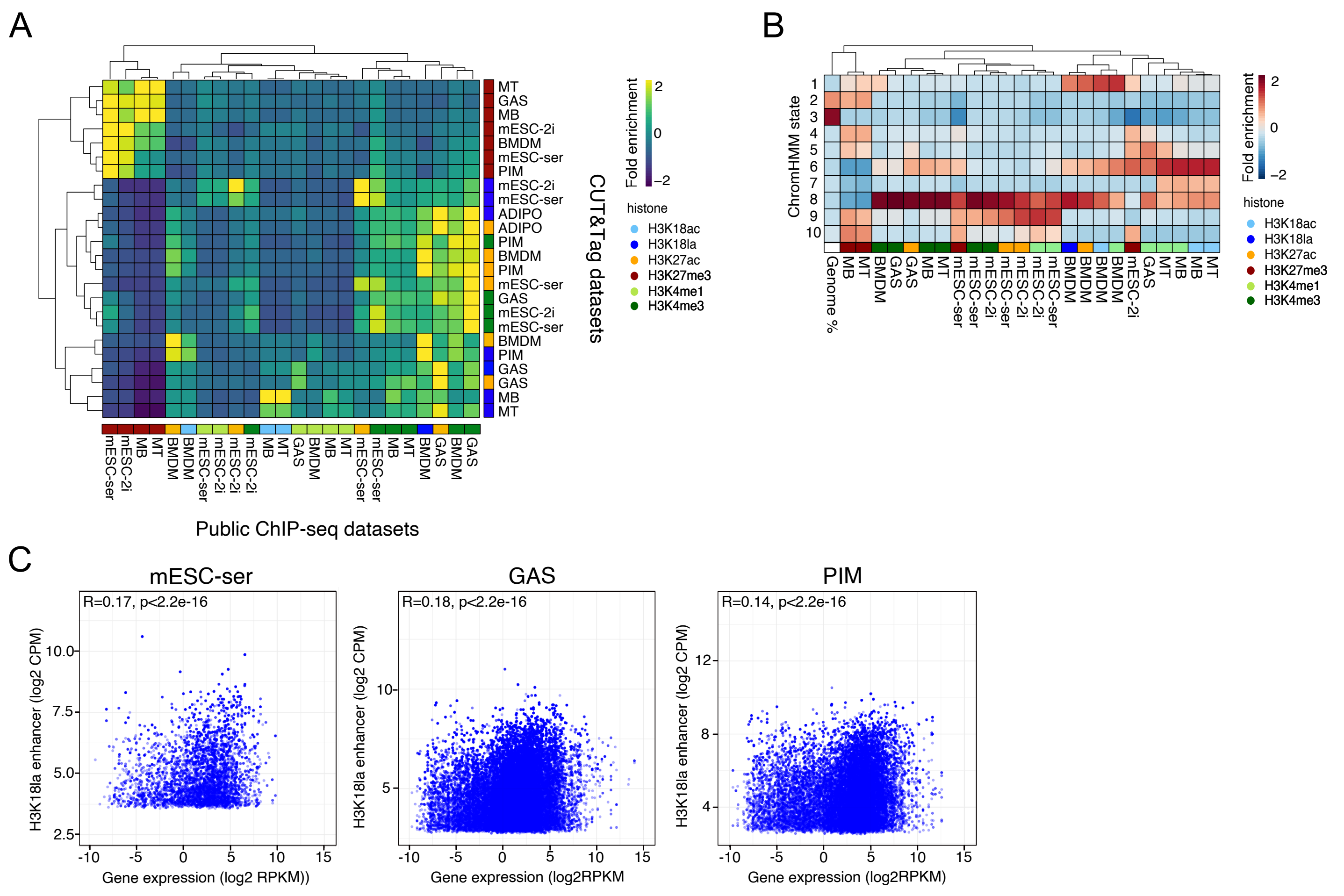
A
Fold enrichment
2
0
−2
histone
H3K18ac
H3K18la
H3K27ac
H3K27me3
H3K4me1
H3K4me3
CUT&Tag datasets
Public ChIP-seq datasets
B
ChromHMM state
Genome %
Fold enrichment
C
mESC-ser
GAS
PIM
R=0.17, p<2.2e-16
R=0.18, p<2.2e-16
R=0.14, p<2.2e-16
H3K18la enhancer (log2 CPM)
Gene expression (log2 RPKM))
Gene expression (log2RPKM
Gene expression (log2RPKM)

Additional File 1: Fig. S4: H3K18la marks active, tissue-specific enhancers.

A) Peak fold enrichment of all included CUT&Tag peak sets (rows) against public ChIPseq peak sets (1–6) (columns). Fold enrichment is calculated as (Σ (bp overlap)/[Σ (bp sample hPTM peaks)*Σ (bp public hPTM peaks)]), normalized per row and scaled from -2 to 2. B) Fold enrichment of ChromHMM states for public ChIPseq hPTM peaks (1–6), scaled from -2 to 2 (see Methods for details). C) Scatter plots depicting H3K18la levels at dELS from ENCODE cCRE peaks (6) (log_2CPM, y-axis) and gene expression of the corresponding closest gene (log_2RPKM, x-axis; see Methods for how dELS were linked to genes). Spearman correlation coefficient R and p-values are indicated.

1. Zhang D, Tang Z, Huang H, Zhou G, Cui C, Weng Y, et al. Metabolic regulation of gene expression by histone lactylation. Nature. 2019 Oct 24;574(7779):575–80.

2. Rovito D, Rerra AI, Ueberschlag-Pitiot V, Joshi S, Karasu N, Dacleu-Siewe V, et al. Myod1 and GR coordinate myofiber-specific transcriptional enhancers. Nucleic Acids Res. 2021 May 7;49(8):4472–92.

3. Asp P, Blum R, Vethantham V, Parisi F, Micsinai M, Cheng J, et al. Genome-wide remodeling of the epigenetic landscape during myogenic differentiation. Proc Natl Acad Sci. 2011 May 31;108(22):E149–58.

4. Perino M, van Mierlo G, Karemaker ID, van Genesen S, Vermeulen M, Marks H, et al. MTF2 recruits Polycomb Repressive Complex 2 by helical-shape-selective DNA binding. Nat Genet. 2018 Jul;50(7):1002–10.

5. Yang P, Humphrey SJ, Cinghu S, Pathania R, Oldfield AJ, Kumar D, et al. Multi-omic Profiling Reveals Dynamics of the Phased Progression of Pluripotency. Cell Syst. 2019 May;8(5):427-445.e10.

6. The ENCODE Project Consortium, Moore JE, Purcaro MJ, Pratt HE, Epstein CB, Shoresh N, et al. Expanded encyclopaedias of DNA elements in the human and mouse genomes. Nature. 2020 Jul 30;583(7818):699–710.

Fig S5

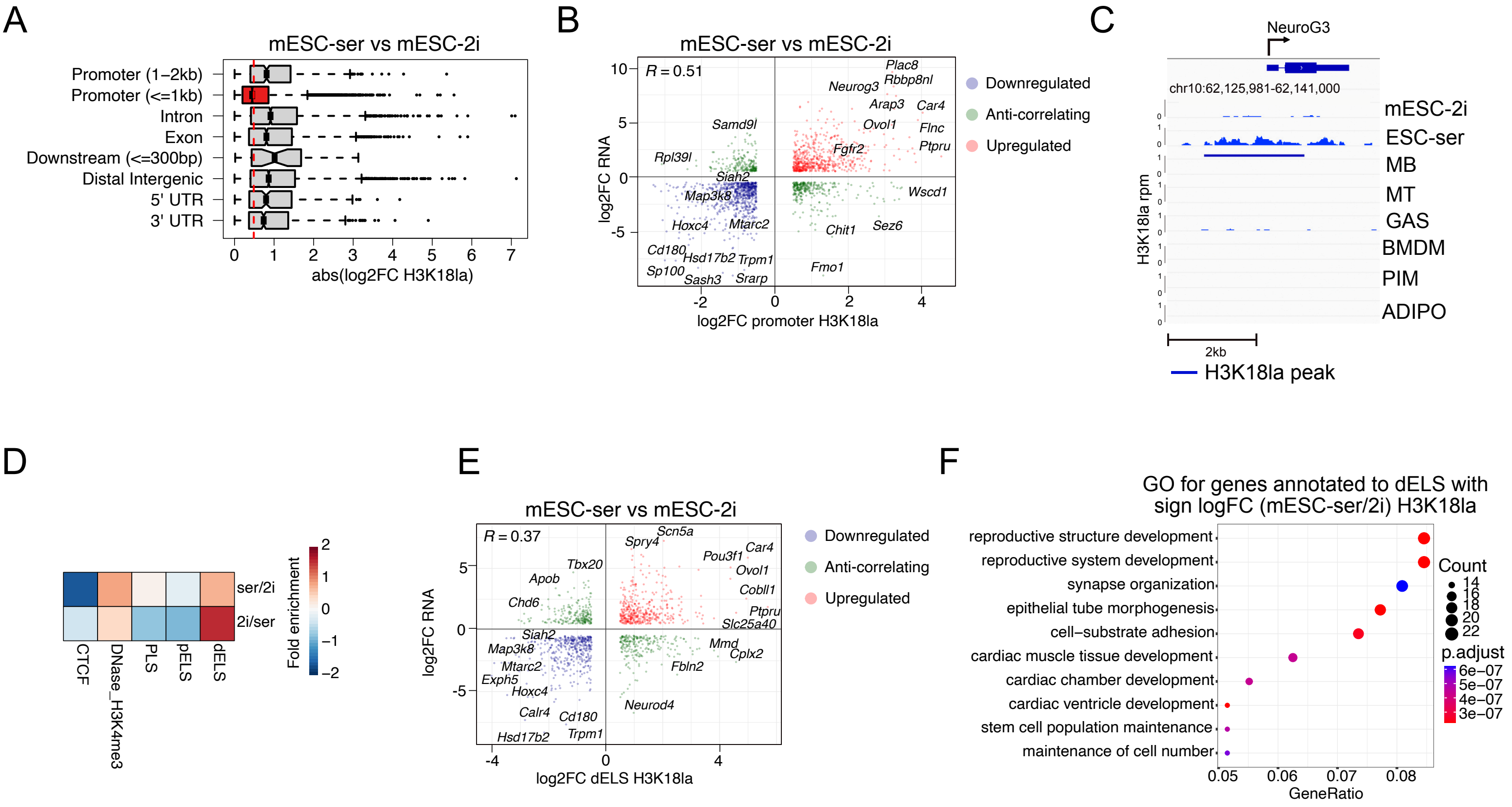
A
mESC-ser vs mESC-2i
Promoter (1–2kb)
Promoter (<=1kb)
Intron
Exon
Downstream (<=300bp)
Distal Intergenic
5' UTR
3' UTR
abs(log2FC H3K18la)
B
mESC-ser vs mESC-2i
R = 0.51
log2FC RNA
log2FC promoter H3K18la
Downregulated
Anti-correlating
Upregulated
C
NeuroG3
chr10:62,125,981-62,141,000
H3K18la rpm
mESC-2i
ESC-ser
MB
MT
GAS
BMDM
PIM
ADIPO
2kb
H3K18la peak
D
ser/2i
2i/ser
CTCF
DNase_H3K4me3
PLS
pELS
dELS
Fold enrichment
E
mESC-ser vs mESC-2i
R = 0.37
log2FC RNA
log2FC dELS H3K18la
Downregulated
Anti-correlating
Upregulated
F
GO for genes annotated to dELS with sign logFC (mESC-ser/2i) H3K18la
reproductive structure development
reproductive system development
synapse organization
epithelial tube morphogenesis
cell−substrate adhesion
cardiac muscle tissue development
cardiac chamber development
cardiac ventricle development
stem cell population maintenance
maintenance of cell number
GeneRatio
Count
p.adjust

Additional File 1: Fig. S5: Dynamic changes of H3K18la reflect transcriptional adaptations in mESC

A) Box plots showing H3K18la log2FC changes from mESC-ser *versus* mESC-2i over different genomic features. B) Scatterplot showing the correlation between significant (FDR < 0.05) H3K18la log_2FC (>0.5) in promoters and their corresponding gene expression log_2FC (>0.5) based on the overlapping genes from the mESC-ser *versus* mESC-2i differential analysis. Pearson correlation coefficient R is indicated. C) IGV (1) genome browser snapshot of H3K18la profiles at the *NeuroG3* promoter region from various mouse samples and the corresponding SEACR-called peak regions. H3K18la level is depicted (rpm). Genomic regions are indicated on the top, as well as RefSeq gene names. D) Fold enrichment of significant differential peaks (FDR < 0.05, $|log_2FC| > 1.5$) in ENCODE cCREs. E) Scatterplot showing the correlation between significant (FDR < 0.05) H3K18la log_2FC (>0.5) in dELS and their closest gene expression log_2FC (>0.5) based on the overlapping genes from mESC-ser vs mESC-2i differential analysis. Pearson correlation coefficient R is indicated. F) Top 10 GO terms (category 'Biological Process') based on the GO analysis of the overlapping upregulated genes in mESC-ser (first quadrant red dots) from Additional File 1: Fig. S5E.

1. Robinson JT, Thorvaldsdóttir H, Winckler W, Guttman M, Lander ES, Getz G, et al. Integrative genomics viewer. Nat Biotechnol. 2011 Jan;29(1):24–6.

Fig S6

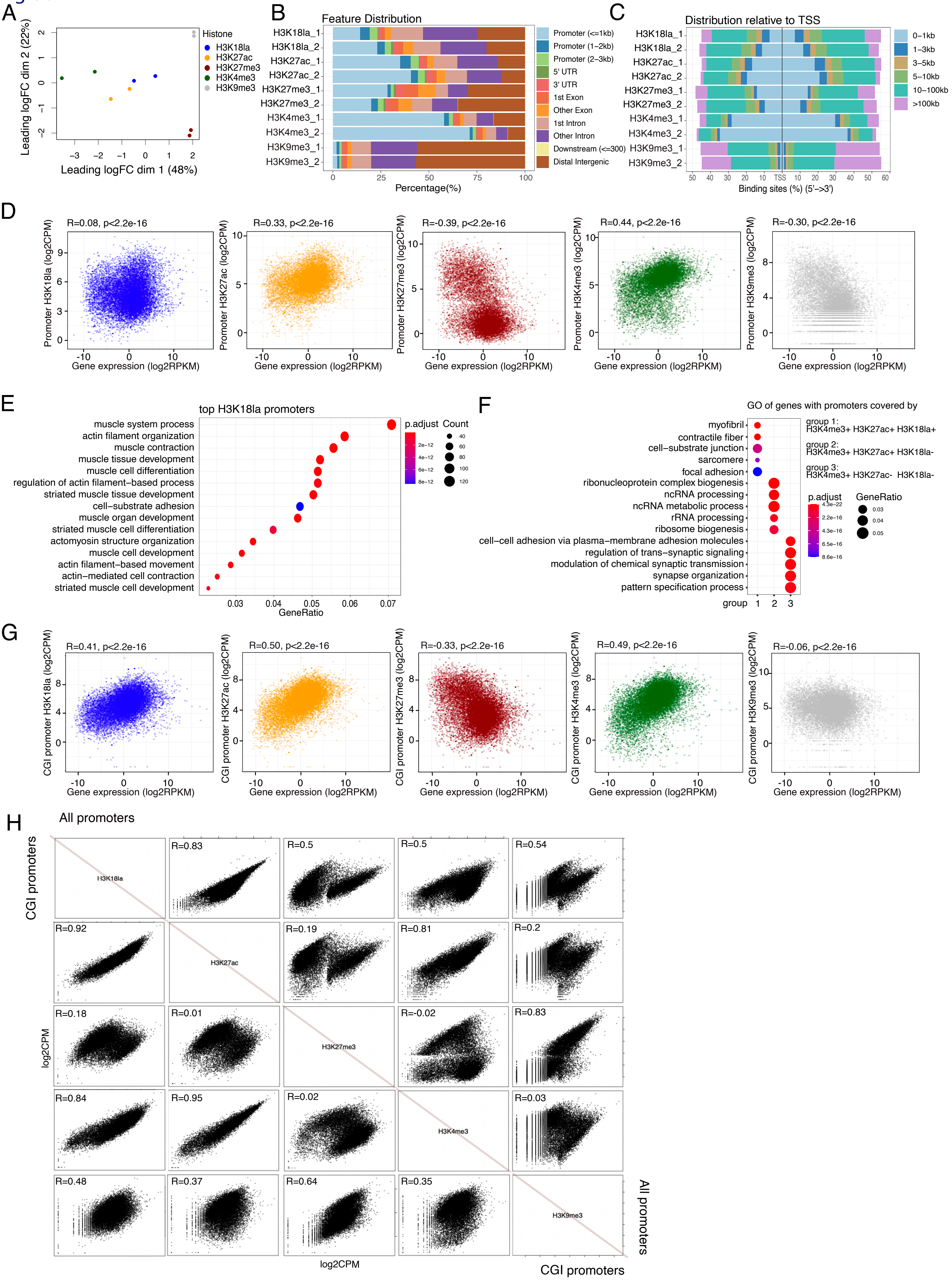
A
Leading logFC dim 2 (22%)
Leading logFC dim 1 (48%)
Histone
H3K18la
H3K27ac
H3K27me3
H3K4me3
H3K9me3
B
Feature Distribution
H3K18la_1
H3K18la_2
H3K27ac_1
H3K27ac_2
H3K27me3_1
H3K27me3_2
H3K4me3_1
H3K4me3_2
H3K9me3_1
H3K9me3_2
Percentage(%)
Promoter (<=1kb)
Promoter (1–2kb)
Promoter (2–3kb)
5' UTR
3' UTR
1st Exon
Other Exon
1st Intron
Other Intron
Downstream (<=300)
Distal Intergenic
C
Distribution relative to TSS
Binding sites (%) (5'->3')
0–1kb
1–3kb
3–5kb
5–10kb
10–100kb
>100kb
D
R=0.08, p<2.2e-16
R=0.33, p<2.2e-16
R=-0.39, p<2.2e-16
R=0.44, p<2.2e-16
R=-0.30, p<2.2e-16
Promoter H3K18la (log2CPM)
Promoter H3K27ac (log2CPM)
Promoter H3K27me3 (log2CPM)
Promoter H3K4me3 (log2CPM)
Promoter H3K9me3 (log2CPM)
Gene expression (log2RPKM)
E
top H3K18la promoters
muscle system process
actin filament organization
muscle contraction
muscle tissue development
muscle cell differentiation
regulation of actin filament–based process
striated muscle tissue development
cell–substrate adhesion
muscle organ development
striated muscle cell differentiation
actomyosin structure organization
muscle cell development
actin filament–based movement
actin–mediated cell contraction
striated muscle cell development
GeneRatio
p.adjust
Count
F
GO of genes with promoters covered by
myofibril
contractile fiber
cell–substrate junction
sarcomere
focal adhesion
ribonucleoprotein complex biogenesis
ncRNA processing
ncRNA metabolic process
rRNA processing
ribosome biogenesis
cell–cell adhesion via plasma–membrane adhesion molecules
regulation of trans–synaptic signaling
modulation of chemical synaptic transmission
synapse organization
pattern specification process
group
group 1: H3K4me3+ H3K27ac+ H3K18la+
group 2: H3K4me3+ H3K27ac+ H3K18la-
group 3: H3K4me3+ H3K27ac- H3K18la-
p.adjust
GeneRatio
G
R=0.41, p<2.2e-16
R=0.50, p<2.2e-16
R=-0.33, p<2.2e-16
R=0.49, p<2.2e-16
R=-0.06, p<2.2e-16
CGI promoter H3K18la (log2CPM)
CGI promoter H3K27ac (log2CPM)
CGI promoter H3K27me3 (log2CPM)
CGI promoter H3K4me3 (log2CPM)
CGI promoter H3K9me3 (log2CPM)
Gene expression (log2RPKM)
H
All promoters
CGI promoters
H3K18la
H3K27ac
H3K27me3
H3K4me3
H3K9me3
R=0.83
R=0.5
R=0.5
R=0.54
R=0.92
R=0.19
R=0.81
R=0.2
R=0.18
R=0.01
R=-0.02
R=0.83
R=0.84
R=0.95
R=0.02
R=0.03
R=0.48
R=0.37
R=0.64
R=0.35
log2CPM
log2CPM
All promoters
CGI promoters

Additional File 1: Fig. S6: Human H3K18la is enriched in active promoters.

A) MDS of all human muscle hPTMs, quantified over 3000 bp genome-wide tiles. B) Distribution of all hPTMs across genomic features for all biological replicates. C) Distribution of hPTM peak distances to TSS for all biological replicates. D) Scatter plots showing the correlation between H3K18la peak levels at promoters (log_2CPM, y-axis) and expression of the corresponding gene (log_2RPKM, x-axis). Spearman's correlation coefficient R and p-values are indicated. E) Top 15 GO terms (category 'Biological Process') resulting from a GO analysis of the genes corresponding to the 2000 promoters with highest H3K18la peaks. F) Comparative GO enrichment analysis (category 'Cellular Component') for genes in group 1 (promoter occupied by H3K4me3+ H3K27ac+ H3K18la), group 2 (promoter occupied by H3K4me3+H3K27ac and not H3K18la) and group 3 (promoter occupied by H3K4me3 and not by H3K27ac nor H3K18la) as defined in Figure 4D. G) Scatter plots showing the correlation between H3K18la peak levels at CGI promoters (log_2CPM, y-axis) and expression of the corresponding gene (log_2RPKM, x-axis). Spearman's correlation coefficient R and p-values are indicated. H) Scatter plots depicting the pairwise correlation of promoter hPTM levels (log_2CPM) at all promoters (upper half) or CGI promoters specifically (lower half). Pearson correlation coefficient R values are indicated.

Fig S7

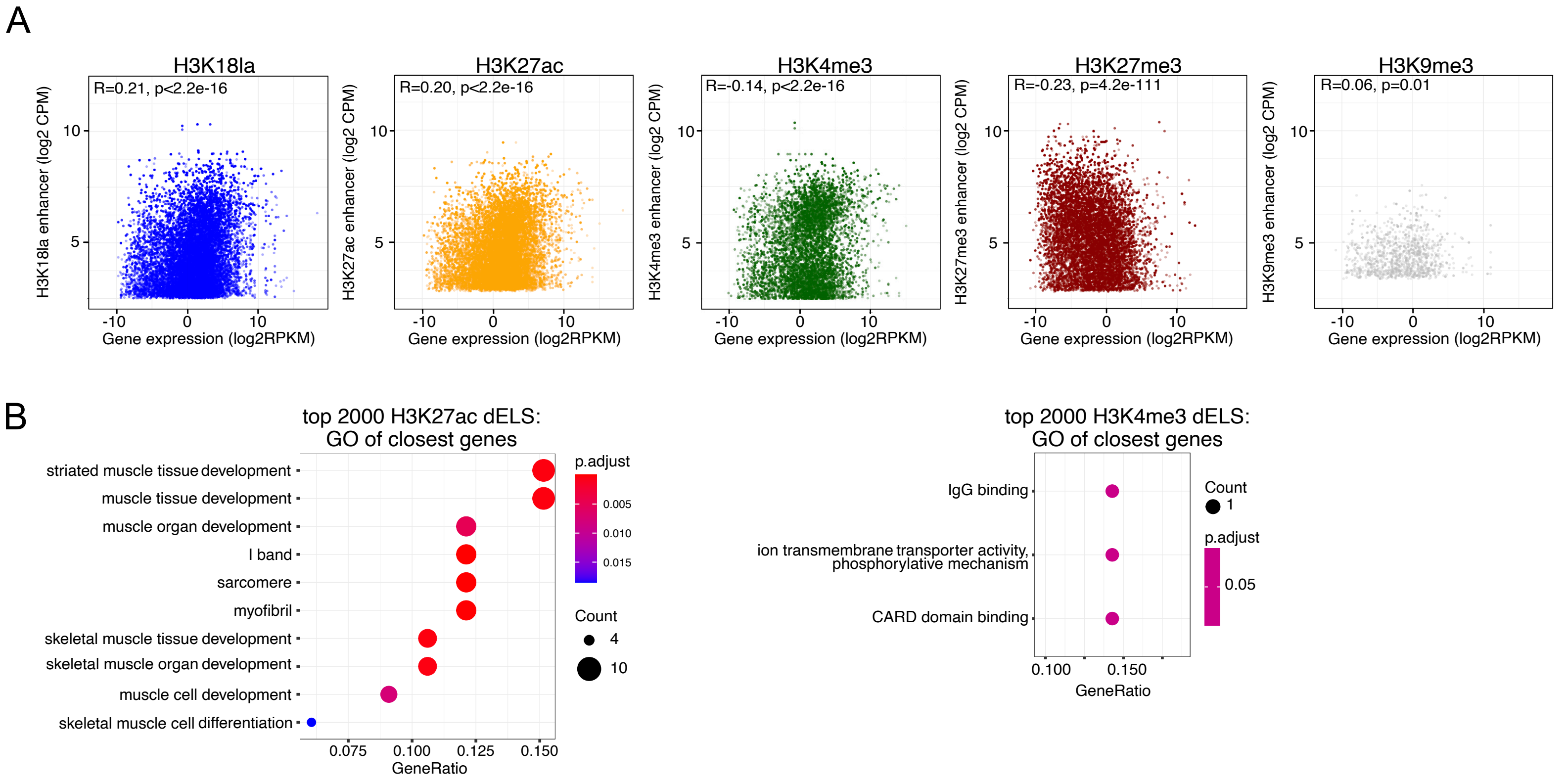
A
H3K18la
R=0.21, p<2.2e-16
H3K18la enhancer (log2 CPM)
Gene expression (log2RPKM)
H3K27ac
R=0.20, p<2.2e-16
H3K27ac enhancer (log2 CPM)
H3K4me3
R=-0.14, p<2.2e-16
H3K4me3 enhancer (log2 CPM)
H3K27me3
R=-0.23, p=4.2e-111
H3K27me3 enhancer (log2 CPM)
H3K9me3
R=0.06, p=0.01
H3K9me3 enhancer (log2 CPM)
B
top 2000 H3K27ac dELS:
GO of closest genes
striated muscle tissue development
muscle tissue development
muscle organ development
I band
sarcomere
myofibril
skeletal muscle tissue development
skeletal muscle organ development
muscle cell development
skeletal muscle cell differentiation
GeneRatio
p.adjust
Count
top 2000 H3K4me3 dELS:
GO of closest genes
IgG binding
ion transmembrane transporter activity,
phosphorylative mechanism
CARD domain binding
GeneRatio
Count
p.adjust

Additional File 1: Fig. S7: Human H3K18la is enriched in active enhancers.

A) Scatter plots depicting peak hPTM levels overlapping with dELS (log_2CPM, y-axis) and gene expression of the corresponding closest gene (log_2RPKM, x-axis; see methods for how dELS are linked to genes). Spearman's correlation coefficient R and p-values are indicated. B) Top GO terms resulting from a GO analysis of the top 2000 genes closest to the enhancers with the highest levels of H3K27ac or H3K4me3.

Fig S8

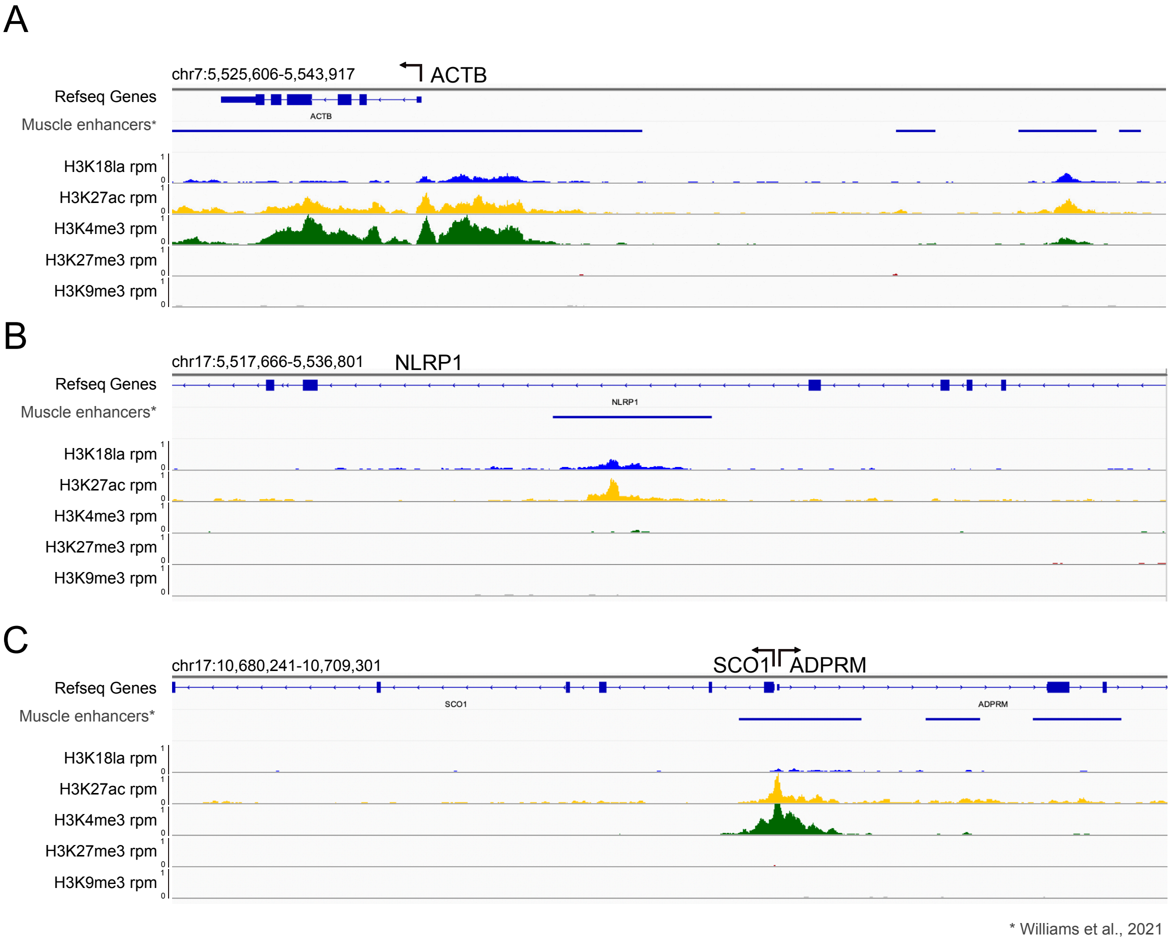
A
chr7:5,525,606-5,543,917
ACTB
Refseq Genes
ACTB
Muscle enhancers*
H3K18la rpm
H3K27ac rpm
H3K4me3 rpm
H3K27me3 rpm
H3K9me3 rpm
B
chr17:5,517,666-5,536,801
NLRP1
Refseq Genes
NLRP1
Muscle enhancers*
H3K18la rpm
H3K27ac rpm
H3K4me3 rpm
H3K27me3 rpm
H3K9me3 rpm
C
chr17:10,680,241-10,709,301
SCO1
ADPRM
Refseq Genes
SCO1
ADPRM
Muscle enhancers*
H3K18la rpm
H3K27ac rpm
H3K4me3 rpm
H3K27me3 rpm
H3K9me3 rpm
* Williams et al., 2021

Additional File 1: Fig. S8: Genome browser (IGV) snapshots of human muscle hPTM profiles.

Genomic regions are indicated on the top as well as human skeletal muscle enhancers (1) and RefSeq Genes. The selected regions include (A) the gene *ACTB* and its flanks, (B) part of the gene *NLRP1*, and (C) TSS and associated first parts of genes *SCO1* and *ADPRM*. H3K18la levels are depicted (rpm).

1. Williams K, Carrasquilla GD, Ingerslev LR, Hochreuter MY, Hansson S, Pillon NJ, et al. Epigenetic rewiring of skeletal muscle enhancers after exercise training supports a role in whole-body function and human health. Mol Metab. 2021 Nov;53:101290.
