# Supplementary material for: H3K18 lactylation marks tissue-specific active enhancers
Source: Genome Biol. 2022 Oct 3;23:207. doi: 10.1186/s13059-022-02775-y (PMC9531456; doi:10.1186/s13059-022-02775-y)

Original Western Blot images from Figure 1A of Galle et al., Genome Biology, 2022

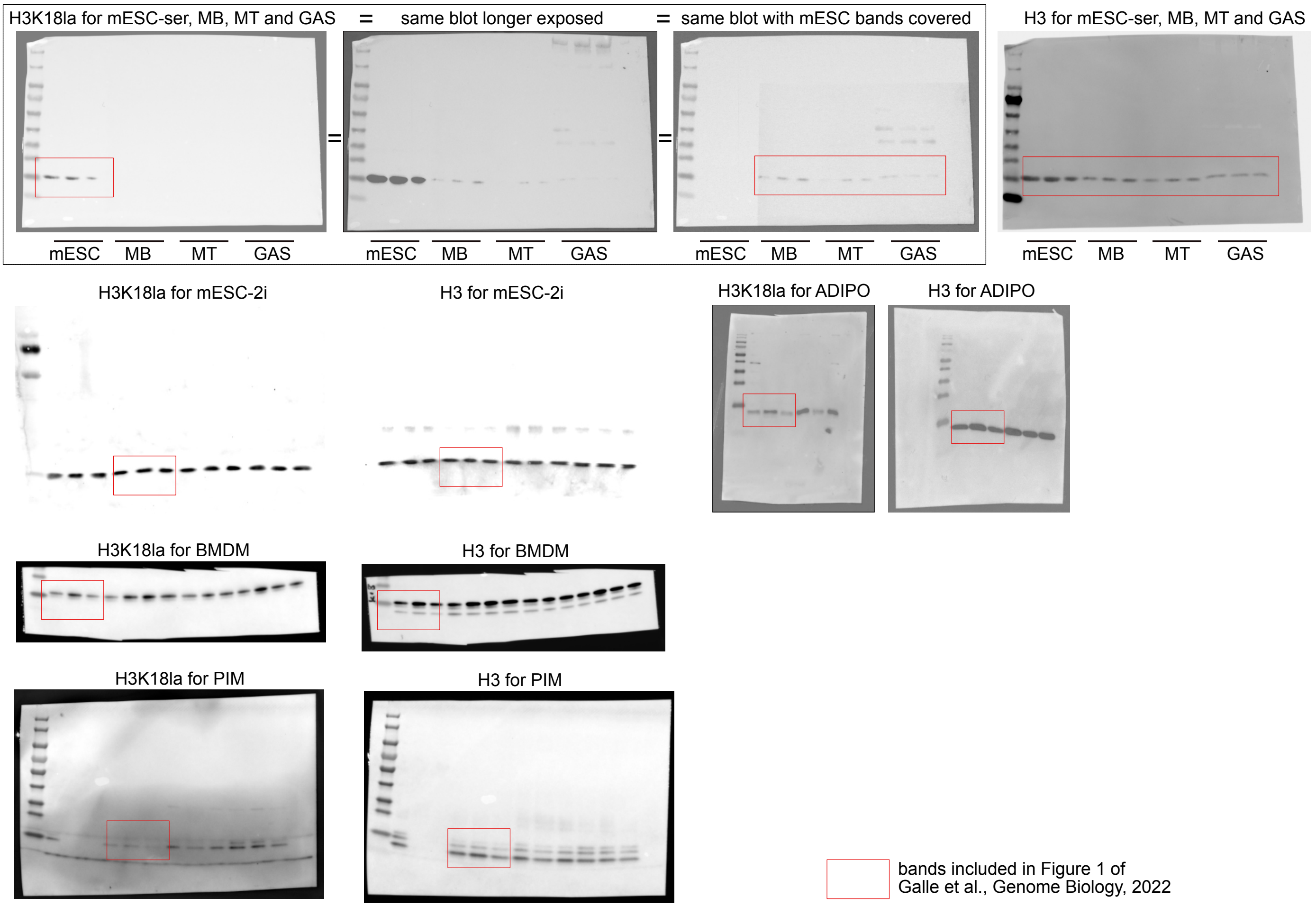

Original Western Blot images from Figure S1B of Galle et al., Genome Biology, 2022

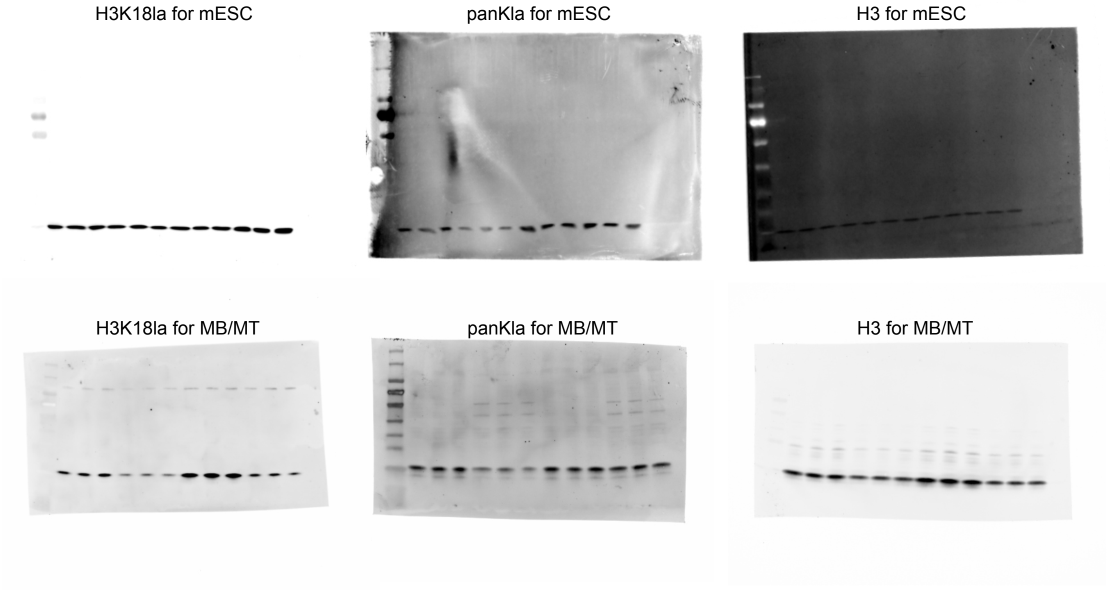

Supplement: Supplementary file 2 — Additional file 2: Uncropped western blot images. Cropped images used in Fig. 1A and Fig S1B. [file 13059_2022_2775_MOESM2_ESM.pdf]
