# Supplementary material for: H3K18 lactylation marks tissue-specific active enhancers
Source: Genome Biol. 2022 Oct 3;23:207. doi: 10.1186/s13059-022-02775-y (PMC9531456; doi:10.1186/s13059-022-02775-y)
Supplement: Supplementary file 7 — Additional file 7. Review history. [file 13059_2022_2775_MOESM7_ESM.docx]

Review History

**First round of review**

**Reviewer 1**

**Were you able to assess all statistics in the manuscript, including the appropriateness of statistical tests used?**

No, I do not feel adequately qualified to assess the statistics.

**Comments to author:**

Histone lactylation was recently described as a novel histone post-translational modification that directly connects cellular metabolism with epigenetic regulation. In this manuscript the authors generated genome-wide datasets of H3K18la distribution in various in vitro and in vivo samples, including embryonic stem cells, macrophages and skeletal muscle and compared them to profiles of well-established histone modifications and gene expression patterns. And shown that H3K18la can marks active promoters and active enhancers.

The genome-wide datasets might be helpful to the field, however, I have the following questions or concerns:

Major points:

1. As we all known, histone lactylation is closely related to cell metabolism, so the metabolic status of the cells used in this manuscript should be described.

2. In this manuscript, differential cell lines such as mESC、MB、MT and GAS were used. These cells differ not only in pluripotency, but also in metabolism. Is the differential distribution of H3K18la due to pluripotency or metabolism?

3. The cell lines selected in this manuscript are all metabolically active cells, and it is suggested to select some cells with low metabolism to demonstrate the universality of the study.

4. As shown in the manuscript, H3K18la can marks active promoters and active enhancers. However, correlation does not necessarily mean causality, as showed in "Histone H3K27 acetylation is dispensable for enhancer activity in mouse embryonic stem cells", so I want to know whether H3K18la directly affects gene expression or enhancer activity? Is it possible to provide some evidence similar to PMID: 33726814 or with other possible strategies?

5. Whether histone acetylation is cross-talked with lactylation? It is suggested to be discussed.

Minor:

1. Language can be simplified in this manuscript, such as "CUT&Tag is a novel alternative technique to ChIP-seq that requires less sample for input and provides cleaner data, with substantial reduced background noise."

2. The data can be presented in a more understandable manner, such as Figure 2A\3E, and the processing method of the data should be shown.

3. ESC is better labeled as mESC.

4. There are two "Figure1" overlapped in page 32.

5. Some labels are lacked in figures, which make the figures more hard to be read. For example, the meaning of color bar in F1D should be labeled in the figure or in the figure legends (better in the figure). F2B, F2D, F3C, F3H, F3I, FS3A and FS3C have similar problems.

6. The scale of genome browser tracks in FS1B-D are lacked. Besides, the meaning of the per-bin score should be noted in the figure legends (RPKM or others?).

**Reviewer 2**

**Were you able to assess all statistics in the manuscript, including the appropriateness of statistical tests used?**

Yes, and I have assessed the statistics in my report.

**Comments to author:**

This manuscript (Galle et al.) presents a rich set of CUT&TAG data for H3K18 lactylation (H3K18la), a novel histone post-translational modification, in multiple human and mouse tissue/cell types, including mouse embryonic stem cells (ESC), primary mouse muscle stem cells (MB), in vitro differentiated mouse myotubes (MT), mouse gastrocnemius muscles(GAS), bone marrow derived mouse macrophages (BMDM) and human vastus lateralis muscles from two individuals. Besides, the CUT&TAG data of several key active/repressive histone marks, including H3K4me3, H3K27ac, H3K27me3 and H3K9me3, in some of these tissue/cell types were also generated, as a reference to dissect the function of H3K18la at different classes of cis regulatory elements. The authors found that H3K18la extensively marks active promoters, showing a strong positive correlation with the H3K4me3 mark and RNA expression level of downstream genes, and also tissue-specific active enhancers in these cells.

However, the computational analyses presented in the current manuscript, in general, seem to be too descriptive, making it difficult to figure out what's the explicit function of H3K18la in transcriptional regulation beyond marking a large subset of active gene promoters and enhancers. To significantly strengthen the manuscript, two fundamental questions should be properly addressed: 1) how the H3K18la level at different classes of regulatory elements is dynamically regulated across cell types; 2) how it is coupled with the cellular metabolic state, considering the claim that it "directly connects cellular metabolism", as well as the histone H3K27ac level, which has also been suggested to be coupled with the cellular metabolism.

Specific comments:

1) It seems that the authors used CPM of RNA-seq reads to quantitate gene transcription levels and correlate them with gene promoter H3K18la levels (Fig.1F). To compare RNA expression level across genes, the RNA-seq read counts should be normalized against gene transcript length. Not sure whether this measure is also used in identifying the genes with the highest and lowest expression levels shown in Fig1E, as it's not mentioned in the Method part.

2) Given the typical size of H3K18la peaks (~2-5kb as shown in genome browser screenshots such as supp fig 1B-D), it's not meaningful to distinguish PLS and pELS when analyzing the enrichment of specific cis regulatory elements in H3K18la peaks (Fig 2A-B, etc.). Do the annotated pELSs have stronger H3K18la signals compared to their flanking regions?

3) Although the authors have shown a strong positive correlation between the gene promoter H3K4me3 and H3K18la levels (Fig. 1), there still seem to be a lot of active gene promoters that are only marked by H3K4me3 but not H3K18la (as indicated by the chromHMM state 3 and 4 shown in Fig 3H). The authors should provide a more detailed analysis to see how & why H3K18la selectively marks only a subset of active gene promoters and what the functional difference is. Given the availability of histone modification and transcriptional factor (TFs) ChIP-seq data in mESCs and other mouse muscle cell types, this analysis can be easily extended to the mouse part.

4) Similarly, the authors have shown that H3K18la strongly co-localize with H3K27ac and H3K18ac globally and specifically on remote enhancers. Do they have any functional difference? For example, are there a lot of enhancers that are only marked by H3K27ac? Besides, Zhang et al. Nature 2019 (https://doi.org/10.1038/s41586-019-1678-1) has shown that the chromatin H3K18la level anti-correlates with H3K18ac. Then how could they co-localize with each other?

5) ChromHMM is not the commonly used method for comparing the chromatin profiles of a protein across cell types and detecting its cell type-specific peaks. It's more meaningful to directly analyze the quantitative changes of H3K18ac levels at gene promoters and enhancers between closely related cell types (eg. mESC vs MB, MB vs MT/GAS, or using a multi-sample comparison, and, more importantly, between mouse muscles before vs. after heavy excises, if possible, to correlate them with the tissue lactate level), and how they correlate with the changes of other histone marks and the cell type specificity of these elements.

**Authors Response**

**Point-by-point responses to the reviewers’ comments:**

Reviewer #1:

comment 1: As we all known, histone lactylation is closely related to cell metabolism, so the metabolic status of the cells used in this manuscript should be described.

*Reply: We thank the reviewer for this comment. We have assessed intra- and extracellular lactate levels in the in vitro cultured mouse systems included in the manuscript and have added these results to the manuscript. In addition, we also used currently available literature to describe the metabolic character of all included mouse cells and tissues. This information is now presented in the first paragraph of the results section.*

comment 2: In this manuscript, differential cell lines such as mESC, MB, MT and GAS were used. These cells differ not only in pluripotency, but also in metabolism. Is the differential distribution of H3K18la due to pluripotency or metabolism?

*Reply: We thank the reviewer for raising this intriguing point. A better understanding of the role of pluripotency and metabolism in epigenetic regulation remains a big challenge to disentangle since metabolic adaptations and changes concur with cellular differentiation and exit from pluripotency. At this point, and despite the various cell types and datasets we have included and analysed here in our presented work (we now also added naïve mESCs), we cannot finally answer this question. But we can conclude that under normal physiological conditions, the global distribution of H3K18la is in the first place defined by cell type and not by the metabolic state.*

*A) Metabolic regulation: We observe differing metabolic rates (more specifically, different intracellular lactate levels), which might contribute to the differential H3K18la distribution across the genome between different cell types. However, it seems improbable that this should be the dominant causal factor and our results indicate that intracellular lactate levels are not directly related to global H3K18la levels. But, a strong lactate challenge, which could represent an extreme physiological stress situation, can increase global histone lactylation levels (Supplemental Figure 1B). Interestingly, gene expression was only modestly affected by a lactate challenge, but many responding genes had H3K18la peaks in their promoters (Figure 3H).*

*B) Pluripotency contribution: Our data suggests that pluripotency, or more specifically, cell lineage or differentiation status is important for global H3K18la distribution differences, as shown in our MDS plots (Figure 1B and Supplemental Figure 1D). Differentiated cell types cluster together on the first dimension of the MDS plot of genome-wide hPTM signals, while mESCs (pluripotent) cluster separately (Figure 1B). Furthermore, ADIPO (known for its low metabolic rate (1)) clusters together with GAS, MB and MT, all mesenchymal-lineage-derived samples, while all hematopoietic cell types (BMDM and PIM) cluster together separately. It is also noteworthy that MB, MT, and GAS, of common myogenic origin, cluster almost indistinctively, although varying substantially in lactate levels, mitotic activity and even in vitro/in vivo origin.*

*Overall, H3K18la always clusters together with other active marks (H3K27ac, H3K4me3) of the same cell type. The same is true with regards to the distribution of H3K18la over genomic features: all differentiated cell types have relatively higher intronic H3K18la enrichment than mESC and promoter enrichment is cell type specific, not correlative to metabolic rate (Figure 1C and Supplemental Figures 1E-F).*

comment 3: The cell lines selected in this manuscript are all metabolically active cells, and it is suggested to select some cells with low metabolism to demonstrate the universality of the study.

*Reply: To address this question, we have generated additional H3K18la CUT&Tag datasets of white adipocytes, a rather metabolic inactive cell type compared to other tissues such as muscle (1), and analysed these in parallel with the other samples. The results are included in the manuscript (Figures 1-2 and Supplemental Figures 1,4). Overall, we do not find a clear distinction based on the metabolic state of the various cell types.*

comment 4: As shown in the manuscript, H3K18la can marks active promoters and active enhancers. However, correlation does not necessarily mean causality, as showed in "Histone H3K27 acetylation is dispensable for enhancer activity in mouse embryonic stem cells", so I want to know whether H3K18la directly affects gene expression or enhancer activity? Is it possible to provide some evidence similar to PMID: 33726814 or with other possible strategies?

*Reply: To our knowledge, no H3K18la specific writer, reader, or eraser has been described so far.As such, it is hard to test and prove a causal relationship between H3K18la and gene expression activation or stimulation. Nevertheless, we agree with the reviewer that this is an important point which could greatly enhance the impact of the paper and we therefore carried out additional experiments to test the possible functionality of H3K18la in driving gene expression. We analysed the changes in H3K18la and gene expression in closely related cell systems to study the role of H3K18la during cell state transitions (mESC-ser vs mESC-2i and MT vs MB). We noted that dynamic changes of H3K18la levels in promoter and dELS can influence gene expression (Figure 3 and Supplemental Figure 5). We also treated MB with lactate since this is known to stimulate myogenic differentiation (2–4) and then asked whether genes with ‘MT-specific promoter H3K18la enrichment’ showed increased gene expression changes. While overall gene expression changes were small, we observed a putative myogeneic differentiation promoting role of H3K18la. Lactate treatment of myoblasts increased the expression of genes that have H3K18la-marked promoters in MT (Figure 3H); these are also significantly enriched in GO terms like ‘(striated) muscle cell differentiation’ and ‘muscle tissue development’. While this does not represent a direct proof or a clear causal relationship, these results support the notion that increased lactylation of promoters can support the increase in gene expression of genes stimulating myogenesis.*

comment 5: Whether histone acetylation is cross-talked with lactylation? It is suggested to be discussed.

*Reply: Thank you for raising this point which we think was very important for our revisions. In order to better address this comment, we generated additional H3K27ac datasets of mESC, GAS, PIM and ADIPO. We have performed an in-depth comparison of H3K18la and H3K27ac (and H3K4me3) to better understand the role and differences of these active marks and specifically how H3K18la differs from H3K27ac. At gene promoters, H3K27ac is often present without H3K18la, but H3K18la almost never without H3K27ac (Figure 1F). The three major co-occurrence patterns at gene promoters are: H3K4me3+H3K27ac+H3K18la (group 1), H3K4me3+H3K27ac (w/o H3K18la; group 2), and H3K4me3 only (group 3; Figure 1F). Overall, these active marks correlate very well with each other (Figure 1G and Supplemental Figure 2A), especially at CGI gene promoters (Supplemental Figure 2B). We found that both H3K27ac and H3K18la have a positive effect on gene expression, with genes whose promoters are marked by H3K4me3+H3K27ac+H3K18la being more highly expressed than genes w/o H3K18la/H3K27ac at their promoter. Genes with group 1 promoters are enriched in tissue-specific GO-terms, especially compared to group 2 or group 3 promoters (Supplemental Figure 3A). These observations in mouse samples were confirmed in human samples (Figures 4D-F and Supplemental Figures 6E-H).*

*Further, H3K27ac is enriched in certain promoter regions w/o H3K18la enrichment, but this is not true for putative enhancer regions. Indeed, ChromHMM states marked by H3K27ac that are highly enriched at dELS are also marked by H3K18la (Figure 2A). Moreover, H3K18la peaks mark more putative enhancers (dELS) than H3K27ac (Figure 2D). These observations were also confirmed in human samples (Figures 4G-I).*

*Overall, our findings show that H3K18la resembles H3K27ac but also has functions and characteristics that are distinct from it.*

minor comments:

Language can be simplified in this manuscript, such as "CUT&Tag is a novel alternative technique to ChIP-seq that requires less sample for input and provides cleaner data, with substantial reduced background noise."

*Reply: We carefully went through the manuscript to improve the understandability and clarify points that might have been not well explained before. We hope that the revised manuscript is now easier to read and understand.*

*The data can be presented in a more understandable manner, such as Figure 2A\3E, and the processing method of the data should be shown.*

*We have significantly revised our figures and paid attention to present the data in a more understandable manner. We have also included a schematic flow chart (Supplemental Figure 1C) to provide an easy overview of the data processing steps. We would like to point out that all our data analysis codes will be available on github.*

ESC is better labeled as mESC.

*Reply: To avoid any confusion, we now refer to mESCs. Since we added a new mESC dataset of mESC grown in 2i/LIF media, we now refer to mESC-ser and mESC-2i.*

There are two "Figure1" overlapped in page 32.

*Reply: That might have been a processing mistake. We are sorry for the inconvenience, and we hope we do not have such a mistake in our revised submission.*

Some labels are lacked in figures, which make the figures more hard to be read. For example, the meaning of color bar in F1D should be labeled in the figure or in the figure legends (better in the figure). F2B, F2D, F3C, F3H, F3I, FS3A and FS3C have similar problems.

*Reply: Thank you for highlighting these issues. We have now corrected the missing labels in the manuscript and figures.*

The scale of genome browser tracks in FS1B-D are lacked. Besides, the meaning of the per-bin score should be noted in the figure legends (RPKM or others?).

*Reply: Thank you for highlighting this issue. We have addressed it in the revised manuscript and figures.*

Reviewer #2

comment 1: How the H3K18la level at different classes of regulatory elements is dynamically regulated across cell types?

*Reply: To address this question, we quantitatively assessed H3K18la changes between 1) mESC cultured in 2i-LIF and serum-LIF medium and 2) MB and MT since these can be considered dynamically changing during cell state transitions. We added a new figure (Figure 3) and supplemental figure (Supplemental Figure 5) to report our findings. Specifically, we found that core-promoter peaks are more stable than non-promoter peaks (Figure 3A and Supplemental Figure 5A) and that changes in H3K18la levels are enriched at dELS (Figure 3D and Supplemental Figure 5D), which is in line with our earlier ChromHMM analysis across different cell types (Figure 2E). Indeed, in this ChromHMM analysis, we found a chromatin state defined by H3K18la in all cell types, enriched at CGI promoters, including housekeeping gene promoters and cell state specific H3K18la chromatin states being enriched at corresponding tissue-specific enhancers. Accordingly, in our quantitative analysis, we found the biggest changes occurred at dELS that lie closest to genes important for cell-state specific gene expression (Figure 3F and Supplemental Figure 5F). Similarly, MT- and MB-specific enhancers were hyper and hypo-lactylated respectively, during the MB-to-MT cell state transition (Figure 3G).*

comment 2: How it is coupled with the cellular metabolic state, considering the claim that it "directly connects cellular metabolism", as well as the histone H3K27ac level, which has also been suggested to be coupled with the cellular metabolism?

*Reply: Thank you for this important comment, which was also raised by reviewer 1 (please also see our answer to comment 1 from reviewer 1 above). Briefly, we have now assessed intra- and extracellular lactate levels in the in vitro cultured mouse systems included in the manuscript and have added these results (Supplemental Figures 1A-B). In addition, we also used currently available literature to describe the metabolic character of all included mouse cells and tissues. This information is now presented in the first paragraph of the results section. Overall, we do not find a strong correlation between the metabolic state and global H3K18la levels. We assume that H3K18la plays an important role to determine cell specificity and is – under normal physiological conditions - only indirectly connected to the metabolic state of the cells.*

specific comment 1:

*Reply: It seems that the authors used CPM of RNA-seq reads to quantitate gene transcription levels and correlate them with gene promoter H3K18la levels (Fig.1F). To compare RNA expression level across genes, the RNA-seq read counts should be normalized against gene transcript length. Not sure whether this measure is also used in identifying the genes with the highest and lowest expression levels shown in Fig1E, as it's not mentioned in the Method part.*

*To address this comment, we now changed RNA CPM units to RPKM units in all our analyses and updated our results and figures accordingly. This is also specified in the methods section (CUT&Tag peak / RNA-seq correlation). Overall, we did not observe significant changes in our results as a consequence of this change.*

specific comment 2:

Given the typical size of H3K18la peaks (~2-5kb as shown in genome browser screenshots such as supp fig 1B-D), it's not meaningful to distinguish PLS and pELS when analyzing the enrichment of specific cis regulatory elements in H3K18la peaks (Fig 2A-B, etc.). Do the annotated pELSs have stronger H3K18la signals compared to their flanking regions?

*Reply: We thank the reviewer for this comment. We had looked at this issue beforehand and - based on the results we summarise for you below – decided to present the data as we did.*

*We opted to include the distinction between pELS and PLS because i) this annotation is based on the publicly available cell type agnostic cCRE classification from ENCODE [PLS regions are defined as promoter like signatures (<200 bp from TSS) marked by high DNase and H3K4me3, whereas pELS regions are enhancer like signatures (<2,000 bp from TSS) marked by high DNase and H3K27ac (low H3K4me3 in addition, if they fall < 200 bp from TSS)] (https://screen.encodeproject.org/) and ii) we found that they do – sometimes only slightly – differ and this could be of interest to the readers (see revised Figure 2A, 3D, 4G for example). In order to remain consistent throughout the manuscript, we decided to use the same annotation for all analyses, i.e. for peak-based as well as for non-peak-based analyses such as ChromHMM. We also took a closer look at how H3K18la peaks overlap with both PLS and pELS. As pointed out by the reviewer, SEACR often calls multiple close by peaks together as a single peak, resulting in broader peaks, as we have also mentioned in the manuscript. It is therefore, likely that the same SEACR called H3K18la peaks do overlap with both PLS and pELS regions, and indeed the majority of the peaks (47-82%) in each mouse cell type of tissue do overlap with both PLS and pELS (Reviewer Figure 1). However, we also found a large number of peaks overlapping only with PLS (3-17%) or pELS (15-41%).*

*We also quantified the H3K18la signals in PLS and pELS, as suggested by the reviewer. The boxplots in Reviewer Figure 2 show the distribution of normalized H3K18la signals across PLS and pELS, highlighting that H3K18la levels are – for every cell type analysed – slightly higher at the core promoters (PLS) than at the flanking regions (pELS). These small but significant (p < 2.2e-16) H3K18la signal differences might be a reflection of the different H3K4me3 levels at PLS and pELS regions, as they are defined by high and low H3K4me3 respectively.*

specific comment 3:

Although the authors have shown a strong positive correlation between the gene promoter H3K4me3 and H3K18la levels (Fig. 1), there still seem to be a lot of active gene promoters that are only marked by H3K4me3 but not H3K18la (as indicated by the chromHMM state 3 and 4 shown in Fig 3H). The authors should provide a more detailed analysis to see how & why H3K18la selectively marks only a subset of active gene promoters and what the functional difference is. Given the availability of histone modification and transcriptional factor (TFs) ChIP-seq data in mESCs and other mouse muscle cell types, this analysis can be easily extended to the mouse part.

*Reply: Thank you for this important observation and please see our response to reviewer 1, comment 5. To complement our prior analyses, we performed a more in-depth analysis of H3K18la, H3K4me3 and H3K27ac differentially marked regions. In summary, we found that, at gene promoters, while H3K27ac is often present without H3K18la, the opposite is not true. The three major co-occurrence patterns at gene promoters are: H3K4me3+H3K27ac+H3K18la (group 1), H3K4me3+H3K27ac (w/o H3K18la; group 2) or H3K4me3 only- (group 3) (Figure 1F). Overall, these active marks correlate very well (Figure 1G and Supplemental Figure 2A) with each other, especially at CGI gene promoters (Supplemental Figure 2B). We found that both H3K27ac and H3K18la have a positive effect on gene expression, with genes with group 1 promoters are more highly expressed than genes w/o H3K18la/H3K27ac at their promoter. Genes with group 1 promoters are enriched in tissue-specific GO-terms, especially when compared to group 2 and 3 promoters (Supplemental Figure 3A). Similar observations were made for both mouse and human samples (Figures 4D-F, Supplemental Figure 6E-H). Together, our results indicate that H3K18la marks only a subset of active promoters, notably those belonging to the highest expressed genes that are important for tissue biology. We incorporated these findings in the promoter sections of the manuscript for the mouse (Figure 1) and human (Figure 4) samples.*

specific comment 4:

Similarly, the authors have shown that H3K18la strongly co-localize with H3K27ac and H3K18ac globally and specifically on remote enhancers. Do they have any functional difference? For example, are there a lot of enhancers that are only marked by H3K27ac? Besides, Zhang et al. Nature 2019 (https://doi.org/10.1038/s41586-019-1678-1) has shown that the chromatin H3K18la level anti-correlates with H3K18ac. Then how could they co-localize with each other?

*Reply: Thank you for these questions. We went back to the original publication of Zhang et al. Nature 2019 to compare it with our results and – upon thorough verification – believe that our data is not in conflict with the publication by Zhang et al.. They showed that during macrophage polarisation, H3K18la and H3K18ac levels in macrophages do not follow the same temporal dynamics (Zhang et al. Figure 3c). Nevertheless, they also show that H3K18la and H3K18ac co-localise in bulk data obtained from the same cell population (Zhang et al. Extended data figure 4o-p) and that gene-specific H3K18la and H3K18ac levels change in the same direction during macrophage polarisation (Zhang et al. Figure 3e). To assess how H3K18la differs from H3K27ac, we used ChromHMM of our human samples, which showed that there are genomic regions marked by H3K27ac but not by H3K18la. To complement this analysis, we generated additional H3K27ac libraries corresponding to several of our mouse samples (mESC-ser, PIM, GAS and ADIPO). We performed the same analysis for our human samples and investigated whether H3K18la and H3K27ac mark the same enhancer sets.*

*We found that all H3K18la-defined chromatin states are also (albeit not to the same level) defined by H3K27ac, while the opposite is not true (Figure 2A). While H3K27ac is enriched in certain promoter regions w/o H3K18la enrichment, this is not true for putative enhancer regions, with all states defined only by H3K18la and H3K27ac being enriched at dELS (Figure 2A).*

*We also found that H3K18la peaks mark more putative enhancers than H3K27ac peaks (Figure 2D). These observations match the ones made for human samples (Figure 4G-I). Overall, these results indicate that H3K18la may have other/additional functionality/characteristics from H3K27ac, specifically at enhancers.*

specific comment 5:

ChromHMM is not the commonly used method for comparing the chromatin profiles of a protein across cell types and detecting its cell type-specific peaks. It's more meaningful to directly analyze the quantitative changes of H3K18ac levels at gene promoters and enhancers between closely related cell types (eg. mESC vs MB, MB vs MT/GAS, or using a multi-sample comparison, and, more importantly, between mouse muscles before vs. after heavy excises, if possible, to correlate them with the tissue lactate level), and how they correlate with the changes of other histone marks and the cell type specificity of these elements.

*Reply: The reviewer rightfully points out that we used ChromHMM in an unconventional way. We now explicitly mention this observation in our manuscript, with the aim of enhancing clarity for the reader. Most importantly, we also performed 1-vs-1 quantitative analyses as suggested by the reviewer (Figure 3 and Supplemental Figure 5). These analyses support the findings of our all H3K18la sample ChromHMM analysis. Specifically, we evaluated H3K18la dynamics during the transition of mESC cultured in 2i-LIF medium to serum-LIF medium and from myoblasts to myotubes. We added a new figure (Figure 3) and supplemental figure (Supplemental Figure 5) to report our findings. Specifically, we found that core-promoter peaks are more stable than non-promoter peaks (Figure 3A, Supplemental Figure 5A) and that changes in H3K18la levels are enriched at dELS (Figure 3D, Supplemental Figure 5D), which is in line with our earlier ChromHMM analysis across all cell types (Figure 2E). Indeed, in this ChromHMM analysis, we found a chromatin state that was marked by H3K18la for all cell types and enriched at CGI promoters, including housekeeping gene promoters. The biggest quantitative H3K18la level changes occurred at dELS that lie closest to genes important for the respective cell-state transitions (Figure 3F, Supplemental Figure 5F). This observation was also suggested by the cell-type-specific H3K18la ChromHMM states (Figure 2E). Similarly, in accordance with the 1-on-1 overlap between cell-type-specific H3K18la ChromHMM states and cell-type-specific enhancers (Figure 2E), we found that MT- and MB-specific enhancers were hyper and hypo-lactylated respectively during the MB-to-MT cell state transition (Figure 3G).*

*In summary, the supervised 1-on-1 quantitative differential analysis we added to the revised manuscript (Figure 3 and Supplemental Figure 5) supports the results from our previous unsupervised all H3K18la sample ChromHMM analysis (Figure 2E) and we think this could be a good inspiration for future studies to expand and popularize the application of ChromHMM further.*

*1. Gallagher D, Belmonte D, Deurenberg P, Wang Z, Krasnow N, Pi-Sunyer FX, et al. Organ-tissue mass measurement allows modeling of REE and metabolically active tissue mass. Am J Physiol-Endocrinol Metab. 1998 Aug 1;275(2):E249–58.*

*2. Ohno, Ando, Ito, Suda, Matsui, Oyama, et al. Lactate Stimulates a Potential for Hypertrophy and Regeneration of Mouse Skeletal Muscle. Nutrients. 2019 Apr 17;11(4):869.*

*3. Tsukamoto S, Shibasaki A, Naka A, Saito H, Iida K. Lactate Promotes Myoblast Differentiation and Myotube Hypertrophy via a Pathway Involving MyoD In Vitro and Enhances Muscle Regeneration In Vivo. Int J Mol Sci. 2018 Nov 19;19(11):3649.*

*4. Willkomm L, Schubert S, Jung R, Elsen M, Borde J, Gehlert S, et al. Lactate regulates myogenesis in C2C12 myoblasts in vitro. Stem Cell Res. 2014 May;12(3):742–53.*

**Second round of review**

**Reviewer 1**

It is pleasant to receive this revision. I commend the authors on their efforts to reply to the suggestions about the additional data requested, figures and language. They have done a thorough job.

**Reviewer 2**

Overall, most of my previous comments have been carefully addressed in the revised manuscript, except for the question whether there are a lot of enhancers that are only marked by H3K27ac. Based on the new H3K27ac profiles generated during the revision, the authors concluded that “H3K18la peaks mark more putative enhancers than H3K27ac” and “these results indicate that H3K18la may have other/additional functionality/characteristics from H3K27ac, specifically at enhancers”. They need to specify what are the other/additional functions for H3K18la at distal enhancers. For examples, as shown in Figure 2D and 4H, there are a lot of putative enhancers that are only marked by H3K18la or H3K27ac. The authors can perform a systematic comparison between these two groups of enhancers, e.g. functional enrichment of downstream genes, TF occupancy, etc., like what they have done to the gene promoters with different combinations of histone marks.

**Authors Response**

**Point-by-point responses to the reviewers’ comments:**

Reviewer #2:

Overall, most of my previous comments have been carefully addressed in the revised manuscript, except for the question whether there are a lot of enhancers that are only marked by H3K27ac. Based on the new H3K27ac profiles generated during the revision, the authors concluded that “H3K18la peaks mark more putative enhancers than H3K27ac” and “these results indicate that H3K18la may have other/additional functionality/characteristics from H3K27ac, specifically at enhancers”. They need to specify what are the other/additional functions for H3K18la at distal enhancers. For examples, as shown in Figure 2D and 4H, there are a lot of putative enhancers that are only marked by H3K18la or H3K27ac. The authors can perform a systematic comparison between these two groups of enhancers, e.g. functional enrichment of downstream genes, TF occupancy, etc., like what they have done to the gene promoters with different combinations of histone marks.

*Reply: We now here include the requested analysis (GO enrichment and TF-binding site enrichment) for H3K18la/H3K27ac-marked dELS, similar to the one we have included in our revised manuscript for various combinations of hPTMs occupation at gene promoters.*

*For both analyses, we defined 3 groups of dELS similar to Figure 2D and Figure 4H of our revised manuscript, i.e. dELS marked by H3K18la and H3K27ac; dELS marked by H3K18la and not H3K27ac; or dELS marked by H3K27ac and not H3K18la.*

*For the gene enrichment analysis, we linked each dELS to its closest gene, retained all unique genes from each group (Supplementary File for Reviewers 2) and performed a comparative GO analysis (using the function clusterCompare of the enrichGO package in R) between the groups (Figure for Reviewers 2A, and Supplementary File for Reviewers 2).*

*For the TF-binding enrichment, we used the coordinates of the dELS of each group as an input to the Cistrome web-application (http://dbtoolkit.cistrome.org/) ‘What factors have a significant binding overlap with your peak set?’ using the settings ‘all peaks in each sample’ and searching for the data type ‘Transcription factor, chromatin regulator’ (Figure for Reviewers 2B).*

*The GO enrichment of dELS occupied by H3K27ac+H3K18la or by H3K18la w/o H3K27ac are similar. The GO enrichment of H3K27ac w/o H3K18la dELS in mESC, PIM and human muscle, however, shows less enrichment for cell-type-specific cellular components (Figure for Reviewers 2A), biological processes and molecular functions (Supplementary File for Reviewers 2). These results agree with our findings described in the manuscript which focus on the cell-type-specificity of H3K18la positioning. A TF-binding site enrichment analysis identified only minor differences between the different groups, and we cannot draw clear conclusions from these.*

*While the results of the GO analysis are interesting and in agreement with the already included analyses, we did not include them into the final manuscript since the results do not show an overall different pattern from our other results. Overall, we are in favour of keeping the manuscript as it is now, but we would be happy to consider including some of the findings presented here in the supplement or change the sentence that “H3K18la peaks mark more putative enhancers than H3K27ac” and “these results indicate that H3K18la may have other/additional functionality/characteristics from H3K27ac, specifically at enhancers” should the editor and reviewer feel that these changes are needed.*

*Figure legends:*

*Figure for Reviewers 2:*

*A) Comparative GO enrichment analysis (category ‘Cellular Component’) for genes in group 1 (closest to dELS occupied by H3K27ac and H3K18la), group 2 (closest to dELS occupied by H3K18la and not H3K27ac) and group 3 (closest to dELS occupied by H3K27ac and not H3K18la). B) Cistrome analysis of promoter regions of genes belonging to group 1, group2, or group 3. The similarity to the public transcription factor binding site datasets is indicated on the y-axis using the GIGGLE score.*

*Supplementary File for Reviewers 2, page 1:*

*Venn diagram of unique genes belonging to group 1 (closest to dELS occupied by H3K27ac and H3K18la), group 2 (closest to dELS occupied by H3K18la and not H3K27ac) and group 3 (closest to dELS occupied by H3K27ac and not H3K18la).*

*Supplementary File for Reviewers 2, page 2:*

*Comparative GO enrichment analysis (category ‘Cellular Component’) for genes in group 1 (closest to dELS occupied by H3K27ac and H3K18la), group 2 (closest to dELS occupied by H3K18la and not H3K27ac) and group 3 (closest to dELS occupied by H3K27ac and not H3K18la).*

*Supplementary File for Reviewers 2, page 3:*

*Comparative GO enrichment analysis (category ‘Biological Process’) for genes in group 1 (closest to dELS occupied by H3K27ac and H3K18la), group 2 (closest to dELS occupied by H3K18la and not H3K27ac) and group 3 (closest to dELS occupied by H3K27ac and not H3K18la).*

*Supplementary File for Reviewers 2, page 4:*

*Comparative GO enrichment analysis (category ‘Molecular Function’) for genes in group 1 (closest to dELS occupied by H3K27ac and H3K18la), group 2 (closest to dELS occupied by H3K18la and not H3K27ac) and group 3 (closest to dELS occupied by H3K27ac and not H3K18la).*
